# Total Synthesis of Acanthodoral Using a Rearrangement Strategy

**DOI:** 10.1021/acs.orglett.3c03717

**Published:** 2024-01-02

**Authors:** Alina Eggert, Karl T. Schuppe, Hazel L. S. Fuchs, Mark Brönstrup, Markus Kalesse

**Affiliations:** † Institute for Organic Chemistry, Leibniz University Hannover, Schneiderberg 1b, 30167 Hannover, Germany; ‡ Helmholtz Centre for Infection Research, Inhoffenstrasse 7, 38124 Braunschweig, Germany

## Abstract

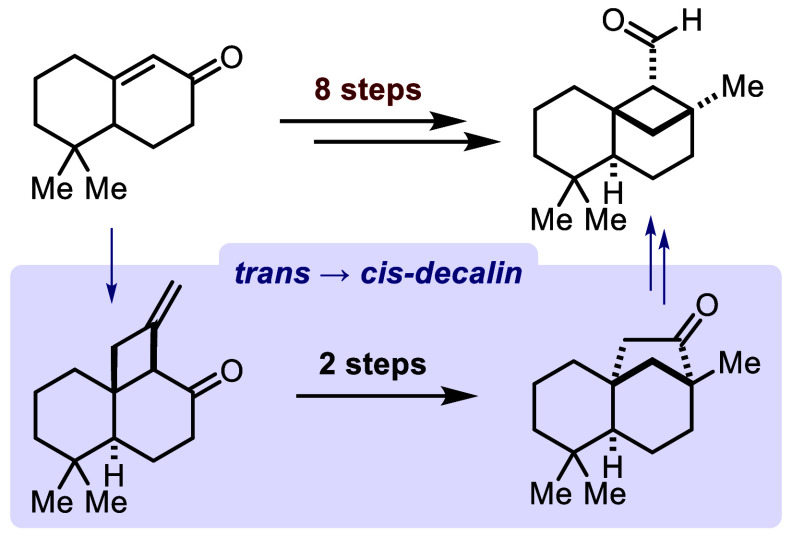

We present the second total synthesis of (±)-acanthodoral, a sesquiterpenoid derived from the marine nudibranch *Acanthodoris nanaimoensis*. Our approach involves a concise three-step transformation from a previously reported compound, resulting in the formation of a less strained precursor of the bicyclo[3.1.1]heptane core and both all-carbon quaternary stereocenters characteristic of the natural product. Notably, this synthetic route incorporates two pivotal steps: a Sm(II)-induced 1,2-rearrangement and a semipinacol rearrangement.

In 1984, Andersen and co-workers isolated the sesquiterpenoid acanthodoral (**1**) along with its two congeners, nanaimoal (**2**) and isoacanthodoral (**3**), from the dorid nudibranch *Acanthodoris nanaimoensis* (Scheme 1a).^1^ This sesquiterpenoid mixture was found to have antibiotic activity against *Bacillus subtilis* and *Staphylococcus aureus* as well as antifungal activity against *Pythiam ultimum* and *Rhizoctonia solani*.^1c^

**Scheme 1:**
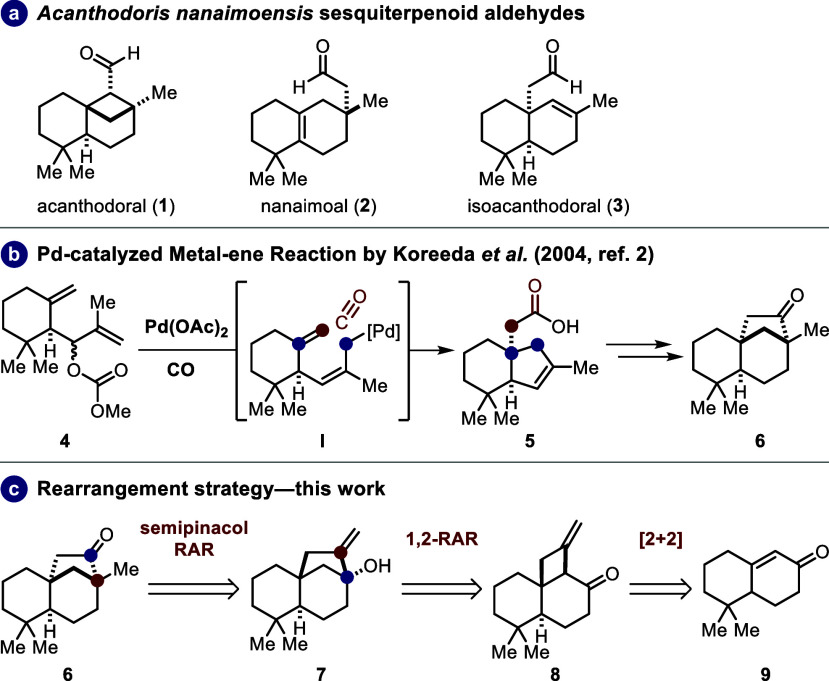
(a) Isomeric Aldehydes from *Acanthodoris nanaimoensis*; (b) Key Step in Koreeda’s Total Synthesis of Acanthodoral (**1**);^2^ (c) Rearrangement Strategy of This Work

All three natural products showcased unique carbon structures,^1^ with the acanthodorane skeleton being particularly noteworthy for synthetic chemists due to its highly strained bicyclo[3.1.1]heptane framework. In a retrosynthetic sense, this bicyclic scaffold is planned to arise from the less strained ketone **6** in an already known sequence from the synthesis of Koreeda and co-workers.^2^ To access the *cis*-decalin core of ketone **6** and the desired stereoconfiguration of its two all-carbon quaternary centers, we envisioned making the semipinacol rearrangement with its stereospecific nature and inherent functional group interconversion. This led us back to methylene alcohol **7**, which can be traced back to cyclobutano ketone **8** through a 1,2-rearrangement. Ultimately, cyclobutano ketone **8** is expected to result from a photochemical [2 + 2] cycloaddition.

The synthesis toward enone **9** starts with commercially available carboxylic acid **10**. Reduction of **10**(3) followed by Appel reaction of the corresponding primary alcohol **11** (not shown) readily afforded bromide **12** in 94% yield over two steps.^4^ Bromide **12** was then exploited as its Grignard reagent to open isobutylene oxide, giving tertiary alcohol **13** in 73% yield.^5^ After treatment with poly(phosphoric acid) and cyclodehydration of **13**, anisole **14** was obtained in 70% yield alongside minor quantities of its *ortho* regioisomer.^6^ Birch reduction^7^ and subsequent acidic hydrolysis of crude diene **15** afforded literature-known enone **9** in 73% yield over two steps (Scheme 2).

**Scheme 2:**
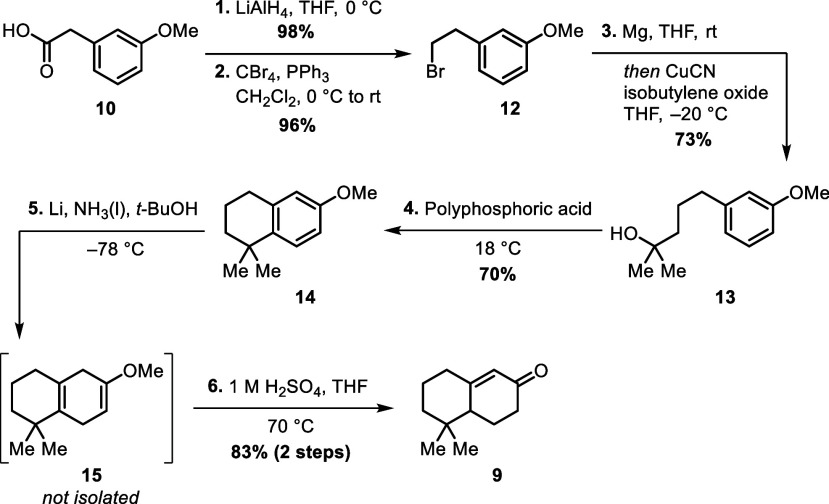
Synthesis of Literature-Known Enone **9**

With enone **9** in hand, we turned our attention to the photochemical [2 + 2] cycloaddition (Scheme 3). Irradiation of enone **9** with condensed allene in methanol at −77 °C readily delivered cyclobutano ketone **8** in 79% yield with a dr of 95:5.^8^ As anticipated, nuclear Overhauser effects of **8** confirmed the [2 + 2] photocycloaddition to result in the more favored *trans*-decalin configuration. To set the stage for the semipinacol rearrangement, cyclobutano ketone **8** first needs to be converted to bicyclo[3.2.1]methylene alcohol **7** in a 1,2-rearrangement. This was put into practice using a method published by Nagaoka and co-workers in 2017.^8^ In a refluxing mixture of SmI_2_, tetra-*n*-butylammonium bromide, and HMPA in THF, cyclopropane species **II** is generated via ketyl–olefin cyclization and fragmentation, of this cyclopropane moiety delivers the bicyclo[3.2.1]heptane core of methylene alcohol **7** in 79% yield (Scheme 3).

**Scheme 3:**
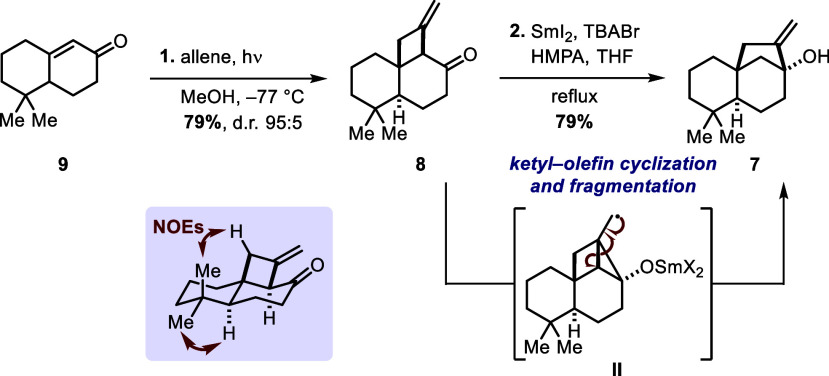
Photochemical [2 + 2] Cycloaddition, Nuclear Overhauser Effects of Cyclobutano Ketone **8**, and Sm(II)-Induced 1,2-Rearrangement TBABr = tetra-*n*-butylammonium bromide; HMPA = hexamethylphosphoramide.

To our delight, subjecting **7** to a refluxing mixture of hydrochloric acid in methanol^9^ sufficiently initiated the envisioned type II semipinacol rearrangement.^10^ Here, the formation of tertiary carbocation **III** can be conceived, which indicates the antiperiplanar alignment of the shifting bond to the unoccupied p orbital, resulting in the desired semipinacol rearrangement pathway to give tricyclic ketone **6** in 76% yield (Scheme 4).

**Scheme 4:**
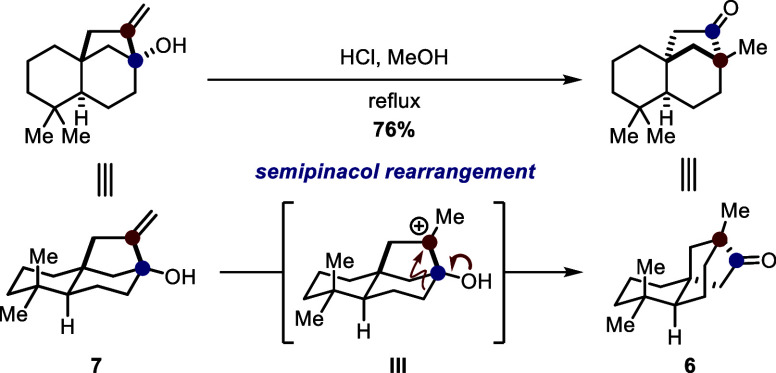
Conditions and Mechanistic Considerations of the Key Semipinacol Rearrangement to Tricyclic Ketone **6**

From semipinacol rearrangement product **6**, the endgame sequence of Koreeda and co-workers^2^ was followed (Scheme 5). To generate the corresponding α-diazo ketone for the ring-contracting Wolff rearrangement, ketone **6** was first treated with KO*t*Bu and isoamyl nitrite at −78 °C to give *syn*-oxime **16** and *anti-*oxime **17** in 79% combined yield (24% *syn*, 55% *anti*). As already described by Koreeda et al., only *anti*-oxime **17** afforded α-diazo ketone **18** under Forster conditions.^11^ By photoirradiation, however, *syn*-oxime **16** could be isomerized to a 43:57 (*syn*:*anti*) mixture in 83% combined yield and was therefore being recycled.

**Scheme 5:**
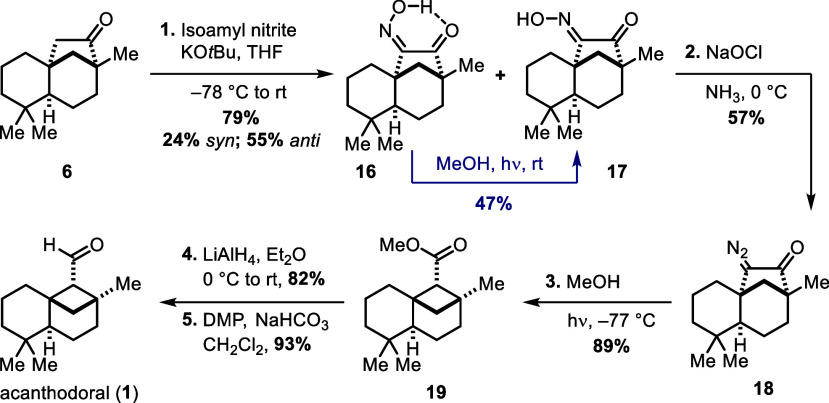
Final Sequence Following the Protocol by Koreeda and Co-workers^2^^,^ DMP = Dess–Martin periodinane.

With α-diazo ketone **18** in hand, irradiation in methanol at −78 °C induced the crucial Wolff rearrangement and afforded methyl ester **19** in 89% yield. Eventually, the reduction of **19** with lithium aluminum hydride and subsequent Dess–Martin oxidation^12^ of the resulting primary alcohol **20** yielded acanthodoral (**1**) in 76% yield over two steps.

Since we strived for a direct way to the natural product and a shorter endgame, we investigated the reduction of the Wolff rearrangement’s intermediary ketene. Here THF was the solvent of choice, as it combines a prolonged lifetime of ketene **IV** and compatibility with reducing agents (Scheme 6). To minimize the risk of overreduction, the use of stoichiometrically defined agents was tested first: While Red-Al gave a mixture of tricyclic primary alcohol **20** and **1**, DIBAL-H delivered **1** as a single product but only in traces (both determined via NMR). On the other hand, the superstoichiometric use of lithium aluminum hydride reliably afforded tricyclic primary alcohol **20** in 40% yield (for details, see the Supporting Information). The reduction of ketene **IV** therefore proved possible, but due to low yields of the natural product or mixtures with primary alcohol **20**, the endgame by Koreeda and co-workers remains more feasible.

**Scheme 6:**
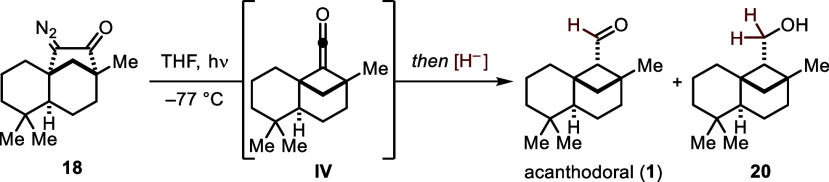
Efforts toward a Shorter Endgame via Reduction of Ketene **IV**

Since the isolation of the pure individual natural products was challenging due to their high volatility and small quantities, **1** and its congeners were isolated as the corresponding (*p*-bromophenyl)urethane derivatives, which also facilitated their structure elucidation.^1^ In order to compare the synthesized **1** to the authentic isolated sample, alcohol **20** was treated with *p*-bromophenyl isocyanate in chloroform at 60 °C to afford carbamate **21** in 65% yield (Scheme 7). Eventually, the NMR spectroscopic data of the synthetic material proved to be in agreement with those published by Andersen and co-workers.^1b^

**Scheme 7:**
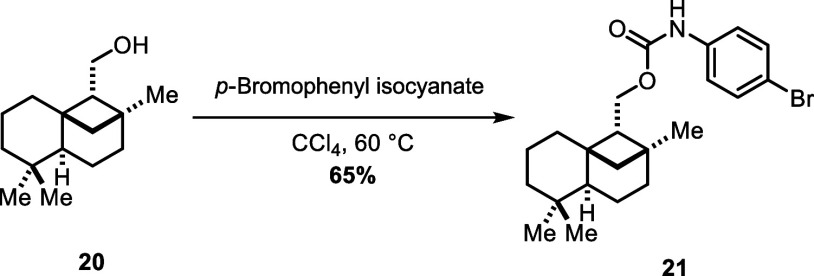
Synthesis of (*p*-Bromophenyl)urethane Derivative **21** for Comparison with Spectral Data of Isolated Material

Lastly, enough material was synthesized to investigate the antimicrobial activity of **1**. Here, primary alcohol **20** and methyl ester **19** were also examined, since they feature the same characteristic bicyclo[3.1.1]heptane core. All three compounds were tested against a selection of bacteria and fungi: *Saccharomyces pombe*, *Mucor hiemalis*, *Candida albicans*, *Bacillus subtilis*, and *Staphylococcus aureus* (see the Supporting Information). The antibiotic activity against *B. subtilis* and *S. aureus* for the isolated sesquiterpenoid mixture (acanthodoral (**1**), nanaimoal (**2**), and isoacanthodoral (**3**))^1c^ was reproduced. Using synthetic **1**, MICs of 106.7 μg/mL were determined against both strains. However, the lability and oxidation sensitivity of **1**, which is readily oxidized to the corresponding carboxylic acid, could lead to an underestimation of its activity. Notably, the aldehyde function was not crucial for antifungal activity, as indicated by the equal or higher potency that alcohol **20** and methyl ester **19** exhibited against *S. pombe* and *M. hiemalis*. Of the three, alcohol **20** proved to be the most potent analog, inhibiting the fungi *S. pombe* and *M. hiemalis* as well as the bacteria *B. subtilis* and *S. aureus* with MICs of 26.6, 13.3, 26.6, and 26.6 μg/mL, respectively.

In conclusion, we accomplished the total synthesis of acanthodoral (**1**) in eight steps (12% yield) from literature-known enone **9** and in 14 steps (4.8% yield) from commercially available carboxylic acid **10**. The semipinacol rearrangement was used to take advantage of the transition from the more easily accessible *trans*-decalin to the sought-after *cis*-decalin of ketone **6**. This provides rapid access (three steps) to the natural product’s two quaternary centers embedded in a less strained precursor of the bicyclo[3.2.1]heptane core. From enone **9**, only two steps—a [2 + 2] photocycloaddition and Sm(II)-induced 1,2-rearrangement—were needed to pave the way for the semipinacol rearrangement. With the direct reduction of the ketene generated by the Wolff rearrangement, a shorter variant of Koreeda’s endgame was explored. However, the endgame reported by Koreeda and co-workers^2^ was eventually followed and remained unchanged. Furthermore, biological assays demonstrate the antifungal and antibacterial activities of the bicyclo[3.1.1]heptanes, which are not dependent on the aldehyde function of **1**.

## Data Availability

The data underlying this study are available in the published article and its Supporting Information.

## Supporting Information Available

Experimental procedures, analytical and spectral data for new compounds, and detailed information on antimicrobial assays (PDF)

## References

[ref1] aAyer S. W.; Hellou J.; Tischler M.; Andersen R. J. Nanaimoal, a Sesquiterpenoid Aldehyde from the Dorid Nudibranch *Acanthodoris nanaimoensis*. Tetrahedron Lett. 1984, 25, 141 10.1016/S0040-4039(00)99824-1.

[ref2] Zhang L.; Koreeda M. Total Synthesis of (+)-Acanthodoral by the Use of a Pd-Catalyzed Metal-ene Reaction and a Nonreductive 5-*exo*-Acyl Radical Cyclization. Org. Lett. 2004, 6, 537–540. 10.1021/ol0363063. 14961617

[ref3] Álvarez-Pérez A.; González-Rodríguez C.; García-Yebra C.; Varela J. A.; Oñate E.; Esteruelas M. A.; Saá C. Catalytic Cyclization of *o*-Alkynyl Phenethylamines via Osmacyclopropene Intermediates: Direct Access to Dopaminergic 3-Benzazepines. Angew. Chem., Int. Ed. 2015, 54, 13357–13361. 10.1002/anie.201505782. 26368394

[ref4] Holmbo S. D.; Pronin S. V. A Concise Approach to Anthraquinone–Xanthone Heterodimers. J. Am. Chem. Soc. 2018, 140, 5065–5068. 10.1021/jacs.8b03110. 29621399

[ref5] Lee J. H.; Deng L. Asymmetric Approach toward Chiral Cyclohex-2-enones from Anisoles via an Enantioselective Isomerization by a New Chiral Diamine Catalyst. J. Am. Chem. Soc. 2012, 134, 18209–18212. 10.1021/ja308623n. 23043531 PMC3492513

[ref6] Parlow J. J. Syntheses of Tetrahydronaphthalenes. Part II. Tetrahedron 1994, 50, 3297–3314. 10.1016/S0040-4020(01)87011-3.

[ref7] Armour A. G.; Büchi G.; Eschenmoser A.; Storni A. Synthese und Stereochemie der Isomeren Ambrinole. Helv. Chim. Acta 1959, 42, 2233–2244. 10.1002/hlca.19590420652.

[ref8] Takatori K.; Ota S.; Tendo K.; Matsunaga K.; Nagasawa K.; Watanabe S.; Kishida A.; Kogen H.; Nagaoka H. Synthesis of Methylenebicyclo[3.2.1]octanol by a Sm(II)-Induced 1,2-Rearrangement Reaction with Ring Expansion of Methylenebicyclo[4.2.0]octanone. Org. Lett. 2017, 19, 3763–3766. 10.1021/acs.orglett.7b01604. 28661153

[ref9] Mander L. N.; Palmer L. T. Synthetic Plant Growth Regulators. IV The Preparation of Hydroxylated Helminthosporic Acid Analogues. Aust. J. Chem. 1979, 32, 823–832. 10.1071/CH9790823.

[ref10] Song Z.-L.; Fan C.-A.; Tu Y.-Q. Semipinacol Rearrangement in Natural Product Synthesis. Chem. Rev. 2011, 111, 7523–7556. 10.1021/cr200055g. 21851053

[ref11] Forster M. O. Azotisation by Chloroamine. J. Chem. Soc. Trans. 1915, 107, 260–267. 10.1039/CT9150700260.

[ref12] Dess D. B.; Martin J. C. Readily accessible 12-I-5 oxidant for the conversion of primary and secondary alcohols to aldehydes and ketones. J. Org. Chem. 1983, 48, 4155–4156. 10.1021/jo00170a070.

