# Supplementary material for: Total Synthesis of Acanthodoral Using a Rearrangement Strategy
Source: Org Lett. 2024 Jan 2;26(15):2893–6. doi: 10.1021/acs.orglett.3c03717 (PMC11041117; doi:10.1021/acs.orglett.3c03717)
Supplement: Supplementary file 1 — ol3c03717_si_001.pdf [file ol3c03717_si_001.pdf]

**Supporting Information for**

# Total Synthesis of Acanthodoral Using a Rearrangement Strategy

Alina Eggert†, Karl T. Schuppe†, Hazel L. S. Fuchs§, Mark Brönstrup§, Markus Kalesse*†

† Institute of Organic Chemistry, Leibniz University Hannover, Schneiderberg 1b, 30167 Hannover (Germany)
§ Helmholtz Centre for Infection Research, Inhoffenstrasse 7, 38124 Braunschweig (Germany)

*E-Mail: markus.kalesse@oci.uni-hannover.de

# Contents

1 General Methods 1

2 Experimental Procedures and Analytical Data 3

3 Ketene Reduction 18

4 Antimicrobial Assays 19

5 References 20

6 Spectra 21

# 1 General Methods

**Performance** All air- and moisture-sensitive reactions were carried out in oven-dried glassware under argon atmosphere with anhydrous solvents using standard Schlenk techniques, sealed with rubber septa and stirred with teflon-coated magnetic stir bars.

**Solvents** Dichloromethane was distilled over calcium hydride under nitrogen atmosphere. THF was distilled over sodium, using benzophenone as an indicator for air and moisture. $Et_2O$ and MeCN, were obtained in Acros AcroSeal™ bottles.

**Reagents** Other reagents and solvents were purchased from commercial suppliers (Acros Organics, Alfa Aesar, Sigma-Aldrich, TCI, abcr) and used without further purification unless otherwise noted. Allene was purchased from abcr.

**Chromatography** Qualitative thin layer chromatography was performed using MACHERY-NAGEL pre-coated silica gel plates on aluminum with fluorescence indicator (0.25 mm silica 60, F254), quantitative thin layer chromatography was performed using Merck pre-coated silica gel plated on glass with fluorescence indicator (0.25 mm silica 60, F254). Visualization was effected by fluorescence detection under UV light at 254 nm or by treating the plate with suitable stain (acidic *p*-anisaldehyde, vanillin, ceric ammonium nitrate or basic $KMnO_4$ stain) and subsequent heating. Flash column chromatography was performed manually using MACHERY-NAGEL silica gel (particle size 40–63 µm).

**NMR Spectroscopy** Nuclear magnetic resonance (NMR) spectroscopy was carried out on the following instruments at room temperature: Bruker Ultrashield 400 MHz (ULS400), Bruker Ascend 400 MHz (ASC400), Bruker Ascend 400 MHz with Prodigy BBFO probe head, Bruker Ultrashield 500 MHz with TCI cryo probe head, Bruker Ascend 600 MHz with DUL cryo probe head. $CDCl_3$ was purchased from commercial suppliers and used as received. The chemical shift $\delta$ is reported in ppm using the residual solvent signal as an internal reference (for $^1H$: $\delta(CHCl_3)$ = 7.26 ppm; for $^{13}C$: $\delta(CDCl_3)$ = 77.16 ppm). Coupling constants $J$ are given in Hz. For multiplicities, the following abbreviations are used: s = singlet; brs = broad singlet; d = doublet; t = triplet; q = quartet; p = pentet; m = multiplet; $m_c$ = centered multiplet and combinations

of these. $^{1}$H-NMR and $^{13}$C{$^{1}$H}-NMR were processed using MestReNova (version 14.2.3) and NOE data were processed using TopSpin (version 3.6.3).

**Mass Spectrometry** High resolution mass spectrometry (HRMS) was performed using a Waters QTof Premier spectrometer with a Waters Acquity UPLC system and electrospray ionization (ESI) as the method of ionization. Gas chromatography was performed using a HP 6890 gas chromatograph with a HP 5973 mass detector and electron ionization (EI) as the method of ionization.

**Photochemical Reactions** All photochemical reactions were performed in flame-dried Quartz glassware. The solvents or reaction mixtures were degassed for 30 min using a sonicator with a pulsed programm. Water-cooled mercury vapour lamps (450 W and 200 W, Hanovia medium pressure, principle emission $\lambda$ = 200 – 400 nm) purchased from Ace Glass Incorp. were used and the Quartz vessel was placed with a distance of 1 cm in front of the UV lamp. Filter glasses were not used. Allene was purchased from abcr.

**Preparation of $SmI_2$-solution (0.1 M in THF)** In a Schlenk tube, samarium granules (7.04 g, 46.8 mmol, 1.2 equiv) were stirred and flame-dried three times under high-vacuum (back-filling with argon, ending under high-vacuum). Meanwhile, excess 1,2-diiodoethane was dissolved in $Et_2O$, washed two times with saturated aqueous $Na_2S_2O_3$-solution, dried over $MgSO_4$, filtered and concentrated under reduced pressure (130 mbar). Under argon, 1,2-diiodoethane (11.0 g, 39.0 mmol, 1.00 equiv) was added to the samarium granules and the Schlenk tube was evacuated and back-filled with argon three times. Then, previously degassed THF (390 ml; 30 min sonicator; distilled, see *Solvents*) was added. To remove the resulting ethylene, the mixture was briefly evacuated and back-filled with argon again. The mixture was stirred at room temperature overnight to give a dark-blue solution.

# 2 Experimental Procedures and Analytical Data

## Primary alcohol 11

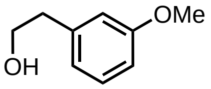

**11**

To a suspension of $LiAlH_4$ (5.71 g, 150 mmol, 2.0 equiv) in dry THF (300 ml, 0.25 M) was added portionwise carboxylic acid **10** (12.5 g, 75.2 mmol, 1.0 equiv) at 0 °C and the resulting reaction mixture was stirred for 5 min at this temperature. Stirring was continued at room temperature for 1 h until the reaction was ended by dropwise addition of water (5.7 ml), following Fieser's workup. Then, 2 M aqueous NaOH (5.7 ml) and water (17.1 ml) were added subsequently and vigorous stirring continued for further 30 min until $Na_2SO_4$ was added. The mixture was filtered and concentration under reduced pressure gave primary alcohol **11** (11.2 g, 73.6 mmol, 98%) as a colorless oil.[1]

**R$_f$** = 0.34 (50% ethyl acetate in petroleum ether)

**HRMS** (ESI) m/z: calcd. for $C_9H_{11}O$ $[M-H_2O+H]^+$ 135.0810, found 135.0810.

**$^1$H-NMR** (400 MHz, $CDCl_3$) $\delta$ = 7.26−7.22 (m, 1H), 6.84−6.81 (m, 1H), 6.80−6.77 (m, 2H), 3.87 (t, 2H, $J = 6.5$ Hz), 3.81 (s, 3H), 2.85 (t, 2H, $J = 6.5$ Hz).

**$^{13}$C{$^1$H}-NMR** (101 MHz, $CDCl_3$) $\delta$ = 159.9, 140.2, 129.7, 121.5, 114.9, 111.9, 63.7, 55.3, 39.4.

*NMR data were identical to those reported in the literature.*[1]

## Primary bromide 12

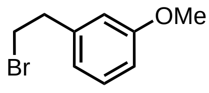


**12**

To a solution of primary alcohol **11** (11.2 g, 73.6 mmol, 1.0 equiv) in $CH_2Cl_2$ (150 ml, 0.5 M) was added at 0 °C $CBr_4$ (26.8 g, 81.0 mmol, 1.1 equiv), followed by slow portionwise addition of $PPh_3$ (21.2 g, 81.0 mmol, 1.1 equiv). After 5 min, the ice bath was removed and stirring was continued for 1 h at room temperature. The reaction mixture was poured over water (200 ml), extracted with EtOAc three times and the combined organic layers were washed with brine. After drying over $MgSO_4$, filtration and concentration under reduced pressure, column chromatography (3% ethyl acetate in petroleum ether) gave primary bromide **12** (15.2 g, 70.6 mmol, 96%) as a colorless oil.[2]

**R$_f$** = 0.93 (50% ethyl acetate in petroleum ether, *p*-anisaldehyde)

**HRMS** (EI) m/z: calcd. for $C_9H_{11}BrO$ [M] 213.9993, found 213.9990.

**$^1$H-NMR** (400 MHz, $CDCl_3$) $\delta$ = 7.26−7.22 (m, 1H), 6.83−6.75 (m, 3H), 3.81 (s, 3H), 3.57 (t, 2H, $J$ = 7.6 Hz), 3.14 (t, 2H, $J$ = 7.6 Hz).

**$^{13}$C{$^1$H}-NMR** (101 MHz, $CDCl_3$) $\delta$ = 159.9, 140.6, 129.8, 121.1, 114.6, 112.3, 55.3, 39.6, 32.9.

*NMR data were identical to those reported in the literature.*[2]

## Tertiary alcohol 13

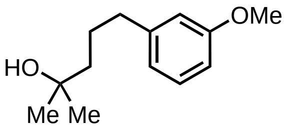

**13**

Tertiary alcohol **13** was prepared using a modification of the protocol reported by Deng and co-workers.[3]

To a solution of activated and freshly mortared magnesium turnings (967 mg, 39.8 mmol, 1.4 equiv) in THF (10 ml, 2.8 M) was slowly added primary bromide **12** (6.11 g, 28.4 mmol, 1.0 equiv) and a water bath was used in case of initial exothermic reaction. The grey solution was stirred for 20 min, while another flask with CuCN (203 mg, 2.27 mmol, 0.1 equiv) in THF (6.0 ml, 0.4 M) was cooled to −20 °C. The Grignard reagent was then dropwise added to the precooled CuCN solution, causing the solution to turn dark. After 1.5 h the solution was warmed to rt, saturated aqueous $NaHCO_3$ solution was added and the mixture was filtered. The phases were separated, the aqueous layer was extracted with $Et_2O$ three times and the combined organic layers were dried over $MgSO_4$, filtered and concentrated under reduced pressure. Column chromatography (55% $Et_2O$ in petroleum ether) gave tertiary alcohol **13** (4.33 g, 20.8 mmol, 73%) as a colorless oil.

**R$_f$** = 0.77 (50% ethyl acetate in petroleum ether, *p*-anisaldehyde)

**HRMS** (EI) m/z: calcd. for $C_{12}H_{15}O$ [M−$H_2O$–Me] 175.1123, found 175.1124.

**$^1$H-NMR** (400 MHz, $CDCl_3$) $\delta$ = 7.22−7.18 (m, 1H), 6.80−6.72 (m, 3H), 3.80 (s, 3H), 2.60 (t, 2H, $J$ = 7.6 Hz), 1.74−1.66 (m, 2H), 1.53−1.49 (m, 2H), 1.21 (s, 6H).

**$^{13}$C{$^1$H}-NMR** (101 MHz, $CDCl_3$) $\delta$ = 159.7, 144.2, 129.4, 121.0, 114.3, 111.1, 71.0, 55.2, 43.6, 36.5, 29.4, 26.3.

*NMR data were identical to those reported in the literature.*[3]

## Bicyclic anisole 14

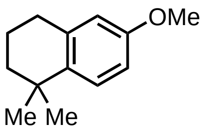

**14**

To mechanically stirred polyphosphoric acid (21.0 g) at 18 °C was dropwise added tertiary alcohol **13** (4.33 g, 20.8 mmol, 1.0 equiv) over 10 min and the reaction mixture started turning red. Stirring continued for 4.5 h until ice water (200 g) was added. The solution was extracted with $Et_2O$ three times, washed with water and brine, dried over $MgSO_4$, filtered and concentrated under reduced pressure. Column chromatography (1% ethyl acetate in petroleum ether) gave bicyclic anisole **14** (2.75 g, 14.5 mmol, 70%) as a colorless oil.[4]

**R$_f$** = 0.43 (100% petroleum ether, vanillin)

**HRMS** (EI) m/z: calcd. for $C_{13}H_{18}O$ [M] 190.1358, found 190.1358.

**$^1$H-NMR** (400 MHz, $CDCl_3$) $\delta$ = 7.26−7.24 (m, 1H), 6.72 (dd, 1H, $J$ = 8.6, 2.8 Hz), 6.58 (d, 1H, $J$ = 2.8 Hz), 3.78 (s, 3H), 2.75 (t, 2H, $J$ = 6.3 Hz), 1.84−1.78 (m, 2H), 1.68−1.64 (m, 2H), 1.27 (s, 6H).

**$^{13}$C{$^1$H}-NMR** (101 MHz, $CDCl_3$) $\delta$ = 157.1, 138.2, 137.5, 127.8, 113.4, 112.3, 55.3, 39.6, 33.4, 32.1, 31.2, 19.9.

*NMR data were identical to those reported in the literature.*[4]

## $\alpha,\beta$-unsaturated enone 9

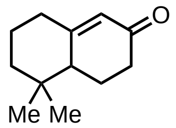


**9**

Crude diene **15** was prepared using a modification of a protocol reported by Storni and co-workers.[5]

In a three necked flask, equipped with a mechanically stirrer, was prepared a solution of lithium (1.16 g, 167 mmol, 5.0 equiv) in liquid ammonia (130 ml) at −78 °C. Then, a solution of anisole **14** (6.38 g, 33.5 mmol, 1.0 equiv) in THF (7.0 ml, 4.8 M) was added and after 20 min, *t*-BuOH (7.8 ml, 81.1 mmol, 2.4 equiv) was added. Stirring was continued for 5 h at this temperature until the reaction was ended through the addition of $NH_4Cl$. The mixture was allowed to warm to room temperature over night to evaporate the ammonia. The resulting slurry was then taken up in MTBE, filtered and concentrated under reduced pressure. Crude diene **15** was then dissolved in THF (112 ml, 0.3 M) and aqueous $H_2SO_4$ (1.0 M, 120 ml) was added. The reaction mixture was stirred at reflux (oil bath) for 4 h. The aqueous phase was extracted with MTBE three times and the combined organic phases were washed with saturated aqueous $NaHCO_3$ solution and brine. After drying over $MgSO_4$, filtration and concentration under reduced pressure, column chromatography (30% MTBE in petroleum ether) gave enone **9** (4.96 g, 27.8 mmol, 83%) as a colorless oil.

**R*f*** = 0.54 (50% $Et_2O$ in petroleum ether, vanillin)

**HRMS** (ESI) m/z: calcd. for $C_{12}H_{18}ONa$ $[M+Na]^+$ 201.1255, found 201.1254.

**$^1$H-NMR** (400 MHz, $CDCl_3$) $\delta$ = 5.90 (s, 1H), 2.47−2.40 (m, 2H), 2.29−2.04 (m, 4H), 1.84−1.59 (m, 3H), 1.53−1.43 (m, 2H), 1.05 (s, 3H), 0.82 (s, 3H).

**$^{13}$C{$^1$H}-NMR** (101 MHz, $CDCl_3$) $\delta$ = 200.1, 166.1, 125.9, 47.3, 41.6, 36.8, 36.1, 35.8, 29.8, 21.9, 21.8, 21.4.

*NMR data were identical to those reported in the literature.*[3]

## Ketone 8

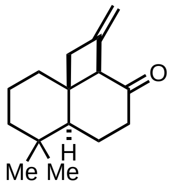


**8**

In a flame-dried Quartz tube, a degassed solution of $\alpha,\beta$-unsaturated enone **9** (350 mg, 1.96 mmol, 1.0 equiv) in MeOH (1.1 ml, 1.8 M) was added to allene (1.5 ml, 26.5 mmol, 14 equiv) at $-77$ °C. After 5 h of irradiation (450 W), the reaction mixture was concentrated under reduced pressure. Column chromatography (10% MTBE in petroleum ether) gave ketone **8** (336 mg, 1.54 mmol, 79%, d.r. 95:5)[1] as a white, amorphous solid.[6]

**$R_f$** = 0.52 (10% EtOAc in petroleum ether, CAN)

**HRMS** (EI) m/z: calcd. for $C_{15}H_{22}O$ [M] 218.1671, found 218.1678.

**$^1$H-NMR** (500 MHz, $CDCl_3$) $\delta$ = 4.99 ($m_c$, 1H), 4.91 ($m_c$, 1H), 3.06−2.98 (m, 2H), 2.69 ($m_c$, 1H), 2.38 ($m_c$, 1H), 2.20 ($m_c$, 1H), 1.95−1.78 (m, 3H), 1.52 ($m_c$, 2H), 1.44−1.35 (m, 2H), 1.28−1.23 (m, 1H), 1.19 (dd, 1H, $J$ = 12.3, 2.9 Hz), 0.94 (s, 3H), 0.78 (s, 3H).

**$^{13}$C{$^1$H}-NMR** (126 MHz, $CDCl_3$) $\delta$ = 210.9, 142.3, 110.2, 63.2, 49.9, 41.8, 40.4, 40.2, 39.7, 37.4, 34.2, 31.6, 20.4, 19.9, 19.4.

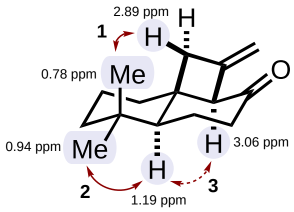


**8**

**For 1D-NOE spectra of NOE contacts 1–3 see pages 34–36.*

[1] The diastereomeric ratio was determined via the $^1$H-NMR of **8**.

## Alcohol 7

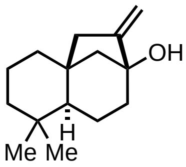

**7**

In a flame-dried flask, $SmI_2$ (0.1 M in THF, 380 ml, 38.0 mmol, 4.0 equiv) was added dropwise to anhydrous TBABr (24.5 g, 75.8 mmol, 8.0 equiv). After refluxing (oil bath) the mixture for 20 min, degassed HMPA (27 ml, 152 mmol, 16 equiv) was added dropwise and refluxing for further 20 min. A degassed solution of ketone **8** (2.07 g, 9.48 mmol, 1.0 equiv) in THF (95 ml, 0.1 M) was added dropwise over 30 min and reflux was maintained for further 3 h. The resulting mixture was cooled to room temperature and the reaction was ended by blowing air into the flask while stirring vigorously. After dilution with $Et_2O$, saturated aqueous $NaHCO_3$ solution and 20% aqueous $NaS_2O_3$ solution (2:1) were sequentially added. The formed precipitate was separated by filtration through Celite® and the filtrate was concentrated under reduced pressure. Column chromatography (7.5% MTBE in petroleum ether) gave alcohol **7** (1.65 g, 7.49 mmol, 79%) as a white, amorphous solid.[6]

**$R_f$** = 0.26 (10% EtOAc in petroleum ether, CAN)

**HRMS** (EI) m/z: calcd. for $C_{15}H_{24}O$ [M] 220.1827, found 220.1821.

**$^1$H-NMR** (400 MHz, $CDCl_3$) $\delta$ = 4.93 ($m_c$, 1H), 4.79 ($m_c$, 1H), 2.73 ($m_c$, 1H), 2.01 ($m_c$, 1H), 1.79−1.60 (m, 5H), 1.43−1.27 (m, 7H), 1.10 (dd, 1H, $J$ = 13.5, 3.8 Hz), 0.88 (s, 3H), 0.87 (s, 3H).

**$^{13}$C{$^1$H}-NMR** (101 MHz, $CDCl_3$) $\delta$ = 157.7, 102.5, 79.6, 56.3, 51.0, 42.6, 40.3, 40.2, 39.9, 39.4, 33.5, 32.9, 22.2, 21.5, 20.0.

## Tricyclic ketone 6

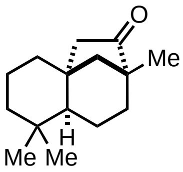

**6**

To a solution of alcohol **7** (130 mg, 0.59 mmol, 1.0 equiv) in MeOH (12.0 ml, 0.05 M) was added HCl (3 M in MeOH, 2.7 ml, 7.97 mmol, 13.5 equiv). The reaction mixture was refluxed (oil bath) for 2.5 h. After cooling to room temperature, water (10 ml) and MTBE (10 ml) were added and the aqueous layer was extracted with MTBE three times. The combined organic layers were washed with saturated aqueous $NaHCO_3$ solution and brine, dried over $MgSO_4$, filtered and concentrated under reduced pressure. Column chromatography (5% MTBE in petroleum ether) gave tricyclic ketone **6** (100 mg, 0.45 mmol, 76%) as a colorless oil.[7]

**$R_f$** = 0.66 (20% EtOAc in petroleum ether, *p*-anisaldehyde)

**HRMS** (EI) m/z: calcd. for $C_{15}H_{24}O$ [M] 220.1827, found 220.1831.

**$^1$H-NMR** (400 MHz, $CDCl_3$) $\delta$ = 2.22 (dd, 1H, $J$ = 18.1, 3.6 Hz), 2.07−2.00 (m, 1H), 1.98−1.91 (m, 1H) 1.81−1.73 (m, 1H), 1.65−1.42 (m, 8H), 1.28−1.18 (m, 3H), 1.04 (s, 3H), 0.99 (s, 3H), 0.92 (s, 3H).

**$^{13}$C{$^1$H}-NMR** (101 MHz, $CDCl_3$) $\delta$ = 223.4, 55.3, 49.6, 49.0, 44.2, 42.2, 40.4, 39.8, 37.1, 34.9, 33.8, 24.3, 20.5, 20.5, 19.6.

*NMR data were identical to those reported in the literature.*[8]

## *syn*-Oxime 16 and *anti*-oxime 17

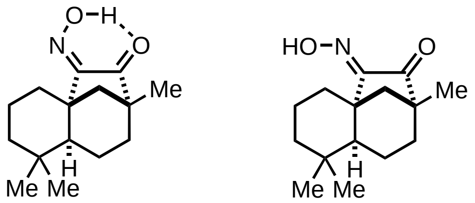


**16** **17**

To a solution of KO*t*-Bu (560 mg, 4.98 mmol, 6.0 equiv) in THF (62 ml, 0.08 M) was added dropwise a solution of tricyclic ketone **6** (183 mg, 0.83 mmol, 1.0 equiv) and isoamyl nitrite (150 µl, 1.25 mmol, 1.5 equiv) in THF (12.0 ml, 0.1 M) at −78 °C. After stirring the reaction mixture at this temperature for 1 h, it was warmed to room temperature and stirred for 1 h . The reaction was ended by addition of $Et_2O$ and saturated aqueous $NH_4Cl$ solution. The aqueous layer was extracted with $Et_2O$ three times and the combined organic layers were washed with brine, dried over $MgSO_4$, filtered and concentrated under reduced pressure. Column chromatography (25% MTBE in petroleum ether) gave *syn*-oxime **16** (51 mg, 0.20 mmol, 24%) as a pale yellow, amorphous solid and *anti*-oxime **17** (115 mg, 0.46 mmol, 55%) as a white, amorphous solid (79% combined yield).[8]

*syn*-Oxime **16** (376 mg, 1.51 mmol, 1.0 equiv) was dissolved in MeOH (15.0 ml, 0.1 M) and degassed. The stirred solution was then irradiated (200 W) at rt for 1 h. The reaction mixture was concentrated under reduced pressure and column chromatography (25% MTBE in petroleum ether) gave two isomeric oximes; *syn*-oxime **16** (134 mg, 0.54 mg, 36%) as a pale yellow, amorphous solid and *anti*-oxime **17** (175 mg, 0.70 mmol, 47%) as a white, amorphous solid.[8]

$\mathbf{R}_f$ = 0.52 (*syn*-oxime **16**) (25% MTBE in petroleum ether, $KMnO_4$)
$\mathbf{R}_f$ = 0.18 (*anti*-oxime **17**) (25% MTBE in petroleum ether, $KMnO_4$)

**HRMS** (*syn*-oxime **16**; ESI) m/z: calcd. for $C_{15}H_{23}NO_2Na$ $[M+Na]^+$ 272.1626, found 272.1629.

**$^1$H-NMR** (*syn*-oxime **16**; 400 MHz, $CDCl_3$) $\delta$ = 13.17 (brs, 1H), 2.36 (d, 1H, $J$ = 12.2 Hz), 1.97−1.21 (m, 12H), 1.09 (s, 3H), 1.06 (s, 3H), 0.94 (s, 3H).

**$^{13}$C{$^{1}$H}-NMR** (*syn*-oxime **16**; 101 MHz, $CDCl_3$) $\delta$ = 210.9, 158.8, 50.1, 48.1, 45.8, 42.2, 41.5, 38.1, 35.3, 34.4, 33.5, 24.1, 20.9, 19.9, 19.9.

**$^{1}$H-NMR** (*anti*-oxime **17**; 400 MHz, $CDCl_3$) $\delta$ = 8.76 (brs, 1H), 2.90 ($m_c$, 1H), 2.36 (d, 1H, $J$ = 12.2 Hz), 1.98−1.94 (m, 1H), 1.82−1.77 (m, 1H), 1.65−1.45 (m, 7H), 1.42−1.32 (m, 2H), 1.11 (s, 3H), 1.08 (s, 3H), 0.96 (s, 3H).

**$^{1}$H-NMR** (*anti*-oxime **17**; 600 MHz, $C_6D_6$) $\delta$ = 10.69 (brs, 1H), 3.10−3.03 (m, 1H), 1.95−1.89 (m, 2H), 1.47−1.16 (m, 9H), 0.99 (s, 3H), 0.88 ($m_c$, 1H), 0.74 (s, 3H), 0.72 (s, 3H).

**$^{13}$C{$^{1}$H}-NMR** (*anti*-oxime **17**; 101 MHz, $CDCl_3$) $\delta$ = 207.9, 160.3, 48.5, 48.3, 44.1, 41.7, 41.5, 36.5, 35.0, 33.9, 33.6, 24.9, 21.5, 20.5, 18.9.

*NMR data were identical to those reported in the literature.*[8]

## Diazo ketone 18

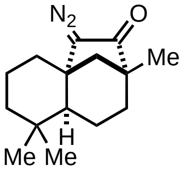


**18**

To a solution of *anti*-oxime **17** (91.5 mg, 367 µmol, 1.0 equiv) in THF (25 ml, 0.015 M) were added $NH_3$ (25% aqueous solution, 4.9 ml) and commercial bleach (4.9 ml) at 0 °C. After 5 min, the reaction mixture was diluted with $Et_2O$ and water was added. The aqueous phase was extracted with $Et_2O$ three times, the combined organic phases were washed with brine, dried over $MgSO_4$, filtered and concentrated under reduced pressure. Column chromatography (17% MTBE in petroleum ether) gave diazo ketone **18** (51.2 mg, 208 µmol, 57%) as a yellow oil.[8]

**R**$_f$ = 0.39 (20% MTBE in petroleum ether, vanillin)

**HRMS** (EI) m/z: calcd. for $C_{15}H_{22}N_2O$ [M] 246.1732, found 246.1731.

**$^1$H-NMR** (400 MHz, $CDCl_3$, 0 °C) $\delta$ = 2.17 (d, 1H, $J$ = 11.4 Hz), 1.82−1.71 (m, 3H), 1.68−1.36 (m, 8H), 1.26−1.19 (m, 1H), 1.04 (s, 3H), 1.03 (s, 3H), 0.95 (s, 3H).

**$^{13}$C{$^1$H}-NMR** (101 MHz, $CDCl_3$, 0 °C) $\delta$ = 203.8, 67.2, 48.7, 46.7, 45.1, 44.9, 41.2, 35.5, 35.1, 34.9, 33.8, 23.9, 20.9, 20.6, 18.9.

*NMR data were identical to those reported in the literature.*[8]

## Tricyclic ester 19

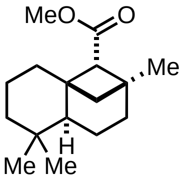

**19**

A degassed solution of diazo ketone **18** (11.0 mg, 45 µmol, 1.0 equiv) in MeOH (15.0 ml, 3 mM) was irradiated (200 W) for 2 h at room temperature. The solution was concentrated under reduced pressure and column chromatography (2.5% MTBE in petroleum ether) gave tricyclic ester **19** (10.0 mg, 40 µmol, 89%).[8]

**R$_f$** = 0.50 (5% MTBE in petroleum ether, vanillin)

**HRMS** (EI) m/z: calcd. for $C_{16}H_{26}O_2$ [M] 250.1933, found 250.1940.

**$^1$H-NMR** (400 MHz, $CDCl_3$) $\delta$ = 3.65 (s, 3H), 2.10 (m$_c$, 1H), 2.02−1.94 (m, 1H), 1.83−1.66 (m, 3H), 1.63−1.31 (m, 7H), 1.20−1.10 (m, 1H), 1.07−1.00 (m, 4H), 0.88 (s, 3H), 0.82 (s, 3H).

**$^{13}$C{$^1$H}-NMR** (101 MHz, $CDCl_3$) $\delta$ = 172.9, 56.5, 50.7, 45.0, 43.1, 41.7, 40.9, 39.5, 38.8, 34.8, 31.7, 31.5, 26.9, 20.9, 19.7, 17.6.

*NMR data were identical to those reported in the literature.*[8]

## Tricyclic primary alcohol 20

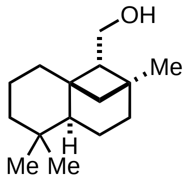

**20**

A solution of tricyclic ester **19** (28.1 mg, 112 µmol, 1.0 equiv) in $Et_2O$ (3.7 ml, 0.03 M) was cooled to 0 °C. Then, $LiAlH_4$ (2.4 M in THF, 75 µl, 179 µmol, 1.6 equiv) was added and the reaction mixture was stirred for 30 min at this temperature and another 30 min at room temperature. After TLC indicated full conversion, the reaction was ended following Fieser's workup. For this, water (2 drops), 15% aqueous NaOH (2 drops) and again water (6 drops) was added. Deviating from Fieser's workup, the aqueous phase was extracted with $Et_2O$ three times and the combined organic phases were washed with brine, dried over $MgSO_4$, filtered and concentrated under reduced pressure. Column chromatography (10% MTBE in petroleum ether) gave tricyclic primary alcohol **20** (20.5 mg, 92 µmol, 82%) as a colorless oil.[8]

**R$_f$** = 0.45 (20% EtOAc in petroleum ether, *p*-anisaldehyde)

**HRMS** (EI) m/z: calcd. for $C_{15}H_{26}O$ [M] 222.1984, found 222.1980.

**$^1$H-NMR** (400 MHz, $CDCl_3$) $\delta$ = 3.65 (d, 2H, $J = 7.3$ Hz), 1.81 (d, 1H, $J = 9.1$ Hz), 1.67−1.30 (m, 11H), 1.10−1.02 (m, 2H), 0.97 (s, 3H). 0.89 (s, 3H). 0.80 (s, 3H).

**$^{13}$C{$^1$H}-NMR** (101 MHz, $CDCl_3$) $\delta$ = 60.3, 56.0, 43.4, 42.1, 42.0, 40.8, 39.3, 37.7, 34.7, 31.4, 30.9, 27.4, 20.7, 19.7, 17.9.

*NMR data were identical to those reported in the literature.*[8]

## Acanthodoral (1)

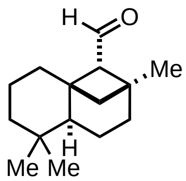

**1**

To a solution of tricyclic primary alcohol **20** (23.0 mg, 104 µmol, 1.0 equiv) in $CH_2Cl_2$ (10.0 ml, 0.01 M) was added $NaHCO_3$ (87 mg, 1.04 mmol, 10 equiv) at 0 °C. After 5 min, Dess–Martin periodinane (66 mg, 156 µmol, 1.5 equiv) was added in one portion. After 1.5 h at this temperature, TLC indicated full conversion and cold *n*-pentane (10 ml) was added. The reaction mixture was then passed through a short plug of silica, which was flushed with $CH_2Cl_2$/*n*-pentane (1:1). Concentration under reduced pressure at 10 °C gave acanthodoral (**1**) (21.3 mg, 97 µmol, 93%) as a pale yellow oil.[8]

**R$_f$** = 0.71 (20% EtOAc in petroleum ether, *p*-anisaldehyde).

**HRMS** (EI) m/z: calcd. for $C_{15}H_{25}O$ [M+H] 221.1905, found 221.1906.

**$^1$H-NMR** (400 MHz, $CDCl_3$, 0 °C) $\delta$ = 9.85 (d, 1H, $J$ = 3.1 Hz), 2.04−1.96 (m, 2H), 1.83−1.47 (m, 7H), 1.45−1.32 (m, 3H), 1.17−1.07 (m, 2H), 1.05 (s, 3H), 0.89 (s, 3H), 0.81 (s, 3H).

**$^{13}$C{$^1$H}-NMR** (101 MHz, $CDCl_3$, 0 °C) $\delta$ = 206.7, 63.4, 46.2, 44.6, 41.4, 41.1, 40.1, 39.5, 35.1, 32.0, 31.4, 27.3, 20.7, 19.5, 17.8.

*NMR data were identical to those reported in the literature.*[8]

## Carbamate 21

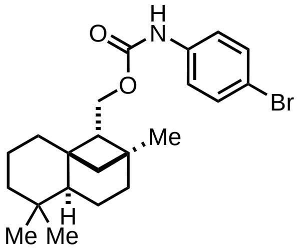


**21**

To a solution of tricyclic primary alcohol **20** (5.2 mg, 23 µmol, 1.0 equiv) in $CCl_4$ (2.0 ml, 0.01 M) was added *p*-bromophenyl isocyanate (19 mg, 97 µmol, 4.2 equiv). The solution was heated to 60 °C (oil bath) and stirred at this temperature for 18 h. After the reaction mixture was cooled to room temperature, MeOH (1 ml) was added and stirring was continued for 1 h. Concentration under reduced pressure and preparative thin-layer chromatography (10% EtOAc in petroleum ether) gave carbamate **21** (6.4 mg, 15 µmol, 65%) as a light-yellow, amorphous solid.[8,9]

$\mathbf{R}_f$ = 0.68 (20% EtOAc in petroleum ether, CAN) .

**HRMS** (ESI) m/z: calcd. for $C_{22}H_{30}NO_2BrNa$ $[M+Na]^+$ 442.1358, found 442.1342.

**$^1$H-NMR** (400 MHz, $CDCl_3$) $\delta$ = 7.41 ($m_c$, 2H), 7.29 ($m_c$, 2H), 6.53 (brs, 1H), 4.16 ($m_c$, 2H), 1.84 (d, 1H, $J = 9.5$ Hz), 1.72−1.30 (m, 11H), 1.11−1.00 (m, 2H), 0.96 (s, 3H), 0.89 (s, 3H), 0.81 (s, 3H).

**$^{13}$C{$^1$H}-NMR** (101 MHz, $CDCl_3$) $\delta$ (of sp$^3$-region)[2] = 63.3, 52.1, 43.3, 42.1, 41.9, 40.8, 38.8, 37.7, 34.7, 31.3, 30.8, 27.1, 20.7, 19.6, 17.7.

*NMR data were identical to those reported in the literature.*[8,9]

[2] Carbon atoms in the aromatic and carbonyl region do not show reliable or evaluable signals in either the $^{13}$C{$^1$H}-NMR or the HSQC spectra.

# 3 Ketene Reduction

**General Procedure** Diazo ketone **18**[1] (1.0 equiv) was dissolved in THF (3 mM) and degassed. The solution was cooled to −78 °C and irradiated (250 W) at this temperature while stirring. After 2 h, irradiation was stopped and the reducing agent was added dropwise. Stirring was continued at −77 °C for further 10 min, before the reaction solution was warmed to room temperature and stirred at this temperature until TLC indicated full conversion (typically 30 min). The reaction was ended by dropwise addition of water. The aqueous phase was extracted with $Et_2O$ and the combined organic phases were washed with brine, dried over $MgSO_4$, filtered and concentrated under reduced pressure.

**Table 3.1** *Reduction of intermediary ketene **IV** with various reducing agents.*

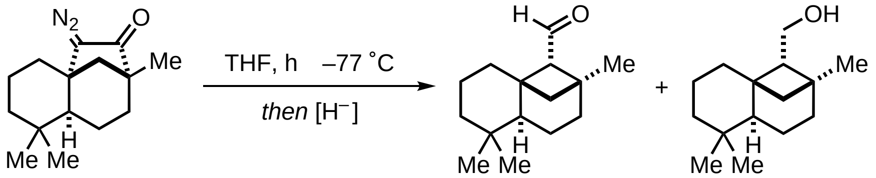


**18** **1** **20**

| entry | reducing agent | equivalents | ratio **1** : **20** | yield |
|---|---|---|---|---|
| 1 | Red-Al® | 1.00 | 1.3:1[a] | –[b] |
| 2 | Red-Al® | 2.00 | 0.8:1[a] | –[b] |
| 3 | DIBAL-H | 1.00 | 95:5[a,c] | traces |
| 4 | $LiAlH_4$ | 6.00 | 5:95[a,c] | 40% |
| 5 | $LiBH_4$ | 8.00 | – | – |

[a] Determined via $^{1}$H-NMR; [b] Purification of mixture not possible due to lability of (**1**); [c] Attributed to $^{1}$H-NMR accuracy).

[1] Usually performed at a scale of about 20 mg.

# 4 Antimicrobial Assays

**General Information** For the tests on antibiotic activity, each substance was dissolved in methanol (stock solution: 6.4 mg/ml) and the following concentrations were used: 426.7 µg/ml, 213.3 µg/ml, 106.7 µg/ml, 53.3 µg/ml, 26.7 µg/ml, 13.3 µg/ml, 6.7 µg/ml, 3.3 µg/ml (with two technical replicates). Methanol was used as a negative control and a known effective antibiotic was used as a positive control. Nystatin was dissolved at 100 µg/ml in methanol and used in antifungal assays at concentrations from 6.67 µg/ml in 2-fold stepwise dilutions until 0.05 µg/ml. For *Bacillus subtilis*, Oxytetracycline was used as a 1 mg/ml stock with dilutions from 66.7 µg/ml to 0.5 µg/ml in the assay. The same dilutions as for Oxytetracycline were used for the control antibiotic Gentamicin, which was the control antibiotic against *Staphylococcus aureus*. The point of growth inhibition was determined by eye and graphs were recorded photometrically ($OD_{600}$).

**Table 4.1** *Tests for antibiotic activity against various bacteria and fungi.*

| | | MIC [$\mu$g/ml] of | | | |
|---|---|---|---|---|---|
| entry | microorganism | acanthodoral (**1**) | alcohol **20** | ester **19** | control |
| 1 | *Saccharomyces pombe*[a] | 53.3 | 26.6 | 53.3 | 2.1[b] |
| 2 | *Mucor hiemalis*[c] | 53.3 | 13.3 | 13.3 | 4.2[b] |
| 3 | *Candida albicans*[d] | >426.6 | >426.6 | >426.6 | 2.1[b] |
| 4 | *Bacillus subtilis*[e] | 106.7 | 26.6 | >426.6 | 16.6[f] |
| 5 | *Staphylococcus aureus*[g] | 106.7 | 26.6 | >426.6 | 0.42[h] |

[a] Type strain DSM70572; [b] Nystatin; [c] Type strain DSM2656; [d] Type strain DSM1655; [e] Type strain DSM10; [f] Oxytetracycline; [g] Type strain DSM346; [h] Gentamicin.

# 5 References


[1] A. Álvarez-Pérez, C. González-Rodríguez, C. García-Yebra, J. A. Varela, E. Oñate, M. A. Esteruelas, C. Saá, *Angewandte Chemie International Edition* **2015**, *54*, 13357–13361.

[2] S. D. Holmbo, S. V. Pronin, *Journal of the American Chemical Society* **2018**, *140*, 5065–5068.

[3] J. H. Lee, L. Deng, *Journal of the American Chemical Society* **2012**, *134*, 18209–18212.

[4] J. J. Parlow, *Tetrahedron* **1994**, *50*, 3297–3314.

[5] A. G. Armour, G. Büchi, A. Eschenmoser, A. Storni, *Helvetica Chimica Acta* **1959**, *42*, 2233–2244.

[6] K. Takatori, S. Ota, K. Tendo, K. Matsunaga, K. Nagasawa, S. Watanabe, A. Kishida, H. Kogen, H. Nagaoka, *Organic Letters* **2017**, *19*, 3763–3766.

[7] L. N. Mander, L. T. Palmer, *Australian Journal of Chemistry* **1979**, *32*, 823–832.

[8] L. Zhang, M. Koreeda, *Org. Lett.* **2004**, *6*, 537–540.

[9] S. W. Ayer, PhD thesis, The University of British Columbia, **1985**.

# 6 Spectra

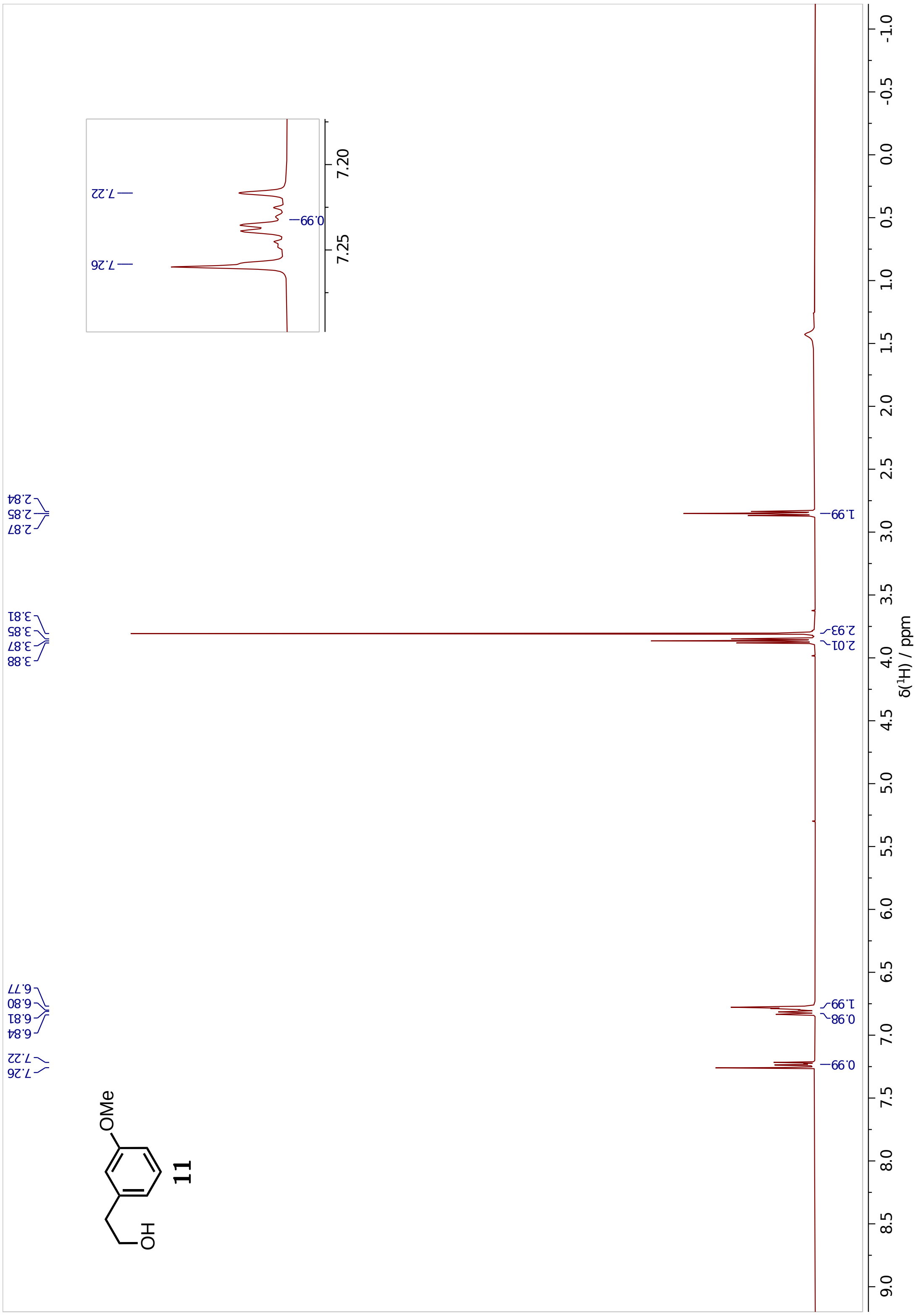


**Spectrum 1** *$^{1}$H-NMR spectrum of substance* ***11*** *measured in $CDCl_3$ at* 400 MHz.

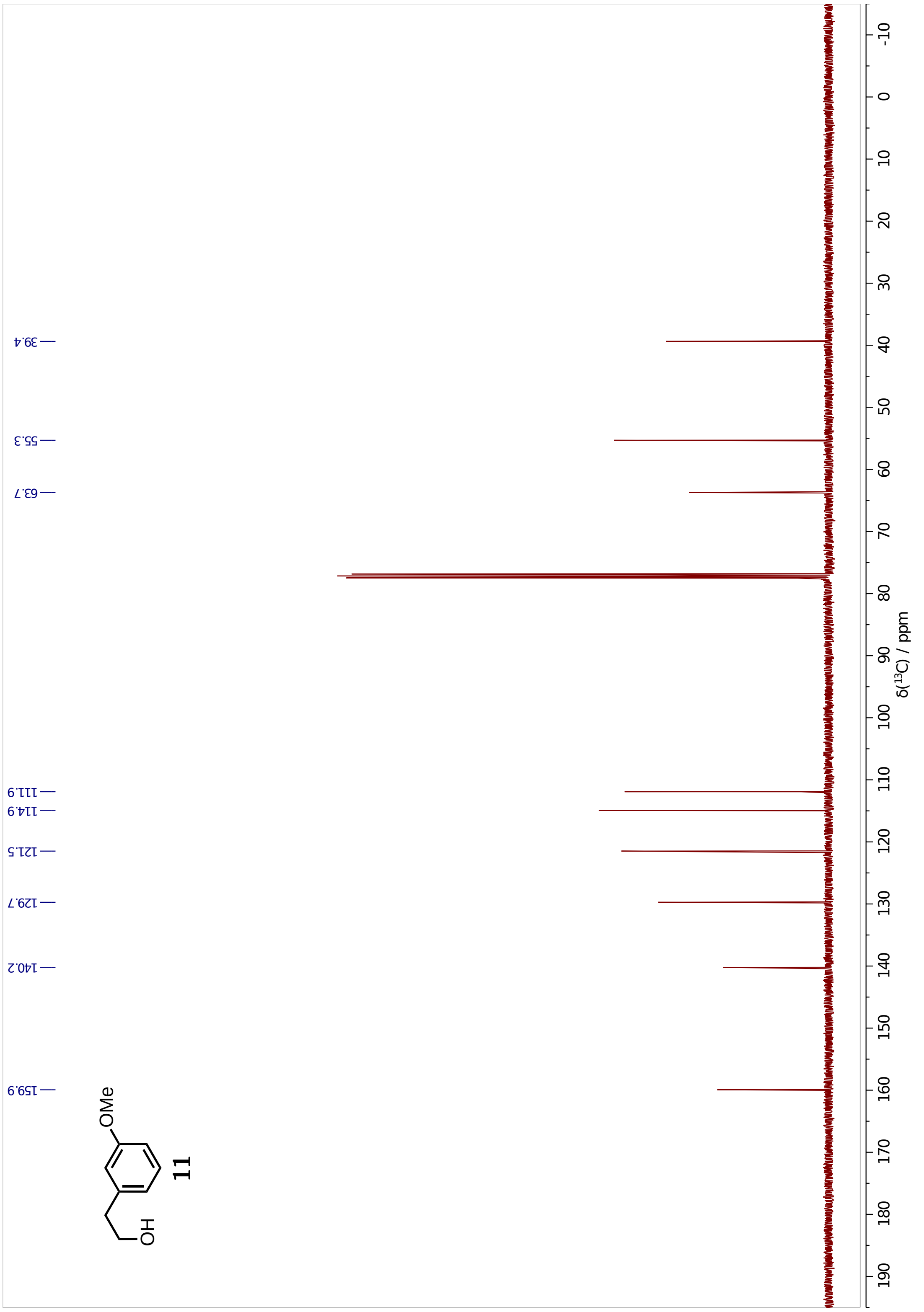


**Spectrum 2** *$^{13}$C-NMR spectrum of substance **11** measured in $CDCl_3$ at* 101 MHz.

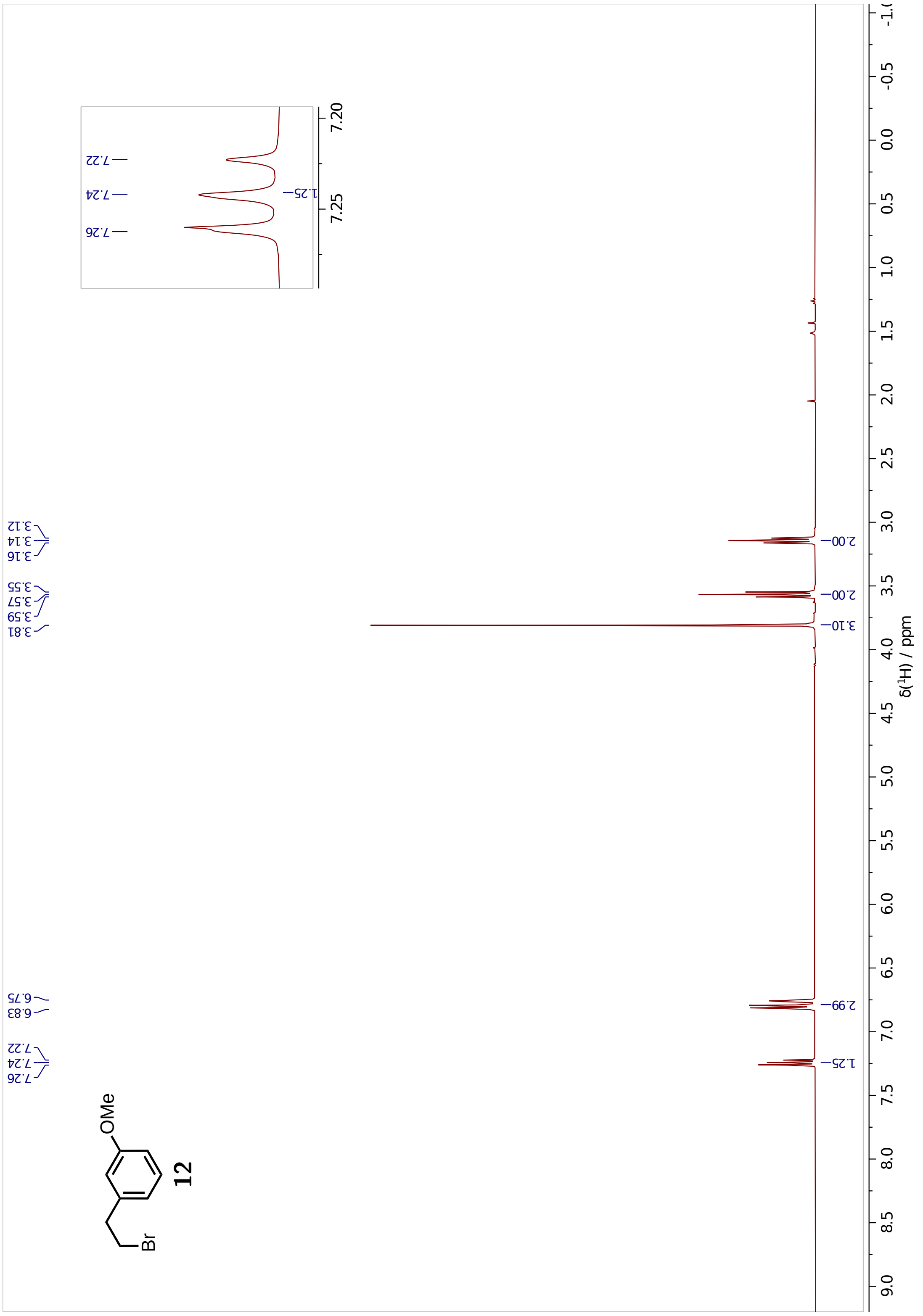


**Spectrum 3** *$^1$H-NMR spectrum of substance **12** measured in $CDCl_3$ at* 400 MHz.

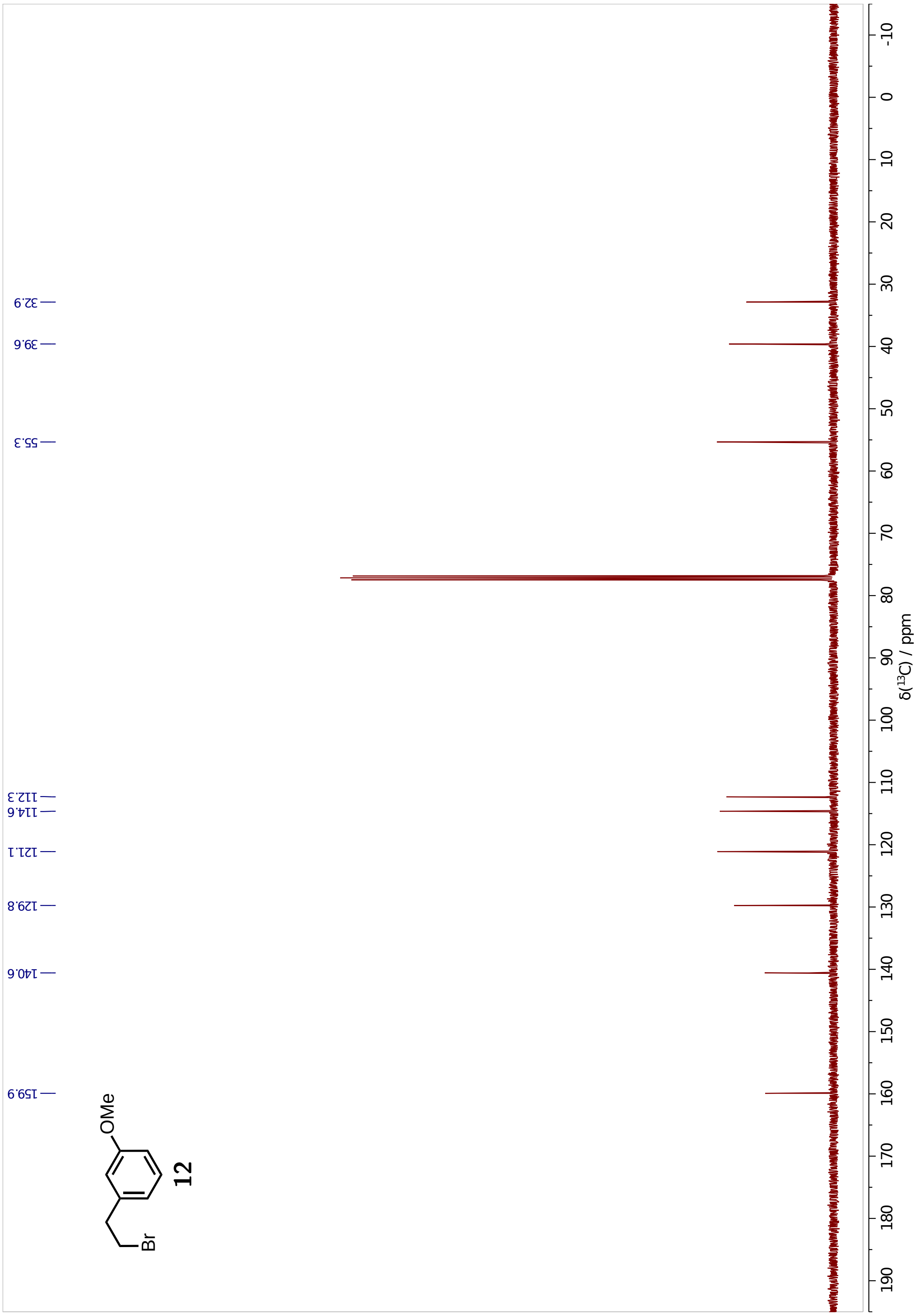


**Spectrum 4** *$^{13}$C-NMR spectrum of substance **12** measured in $CDCl_3$ at* 101 MHz.

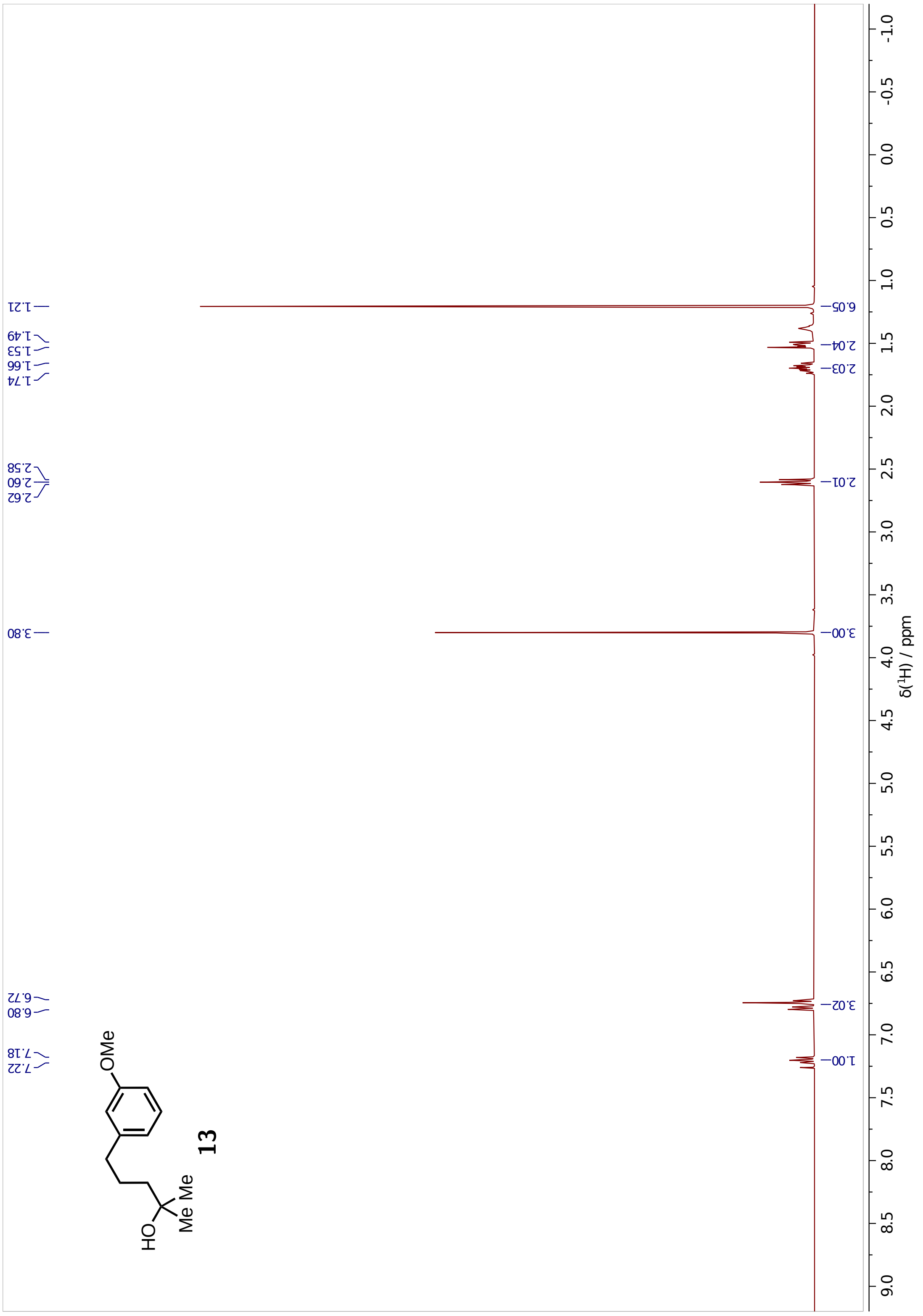


**Spectrum 5** *$^{1}$H-NMR spectrum of substance **13** measured in $CDCl_3$ at* 400 MHz.

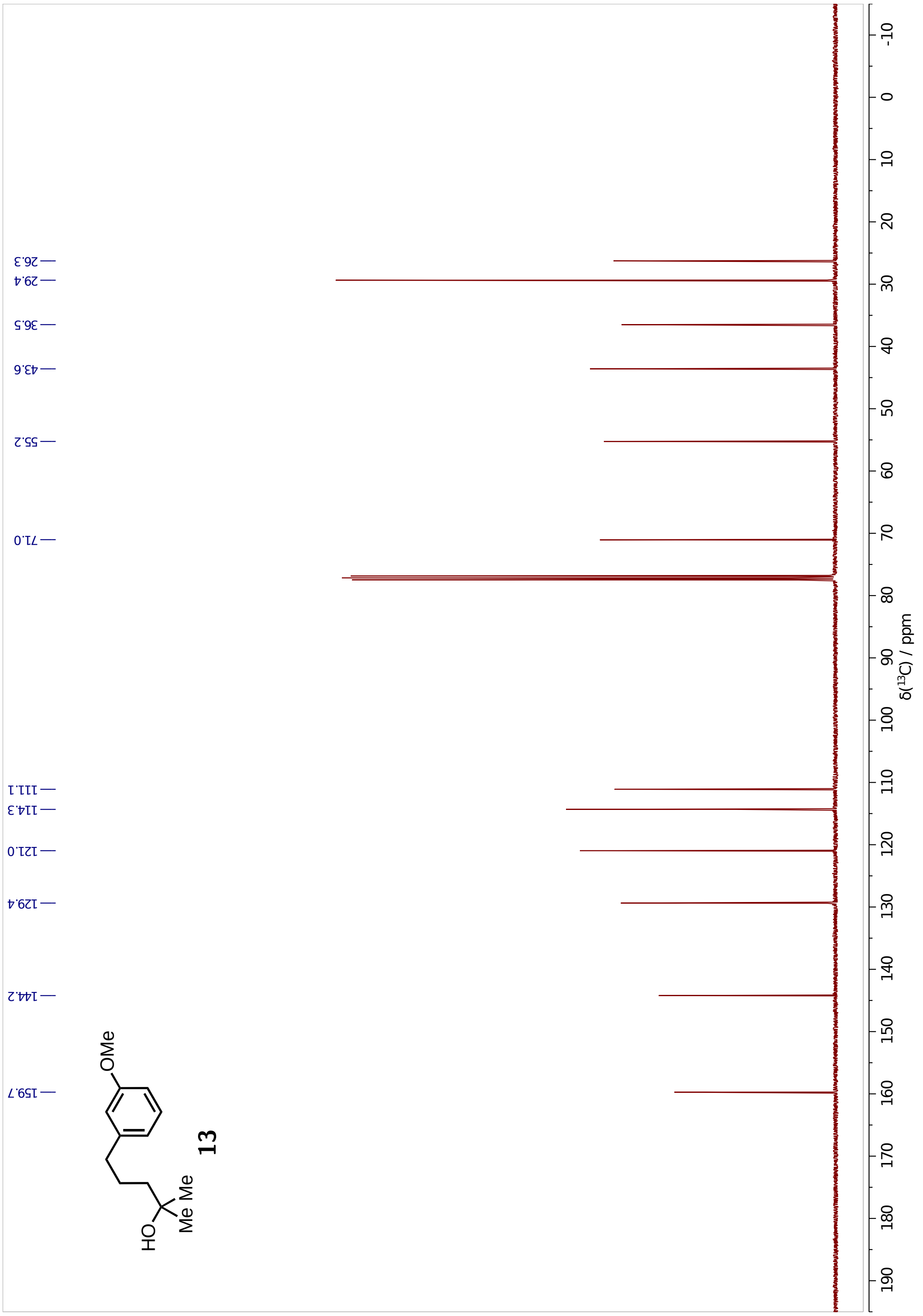


**Spectrum 6** *$^{13}$C-NMR spectrum of substance **13** measured in $CDCl_3$ at* 101 MHz.

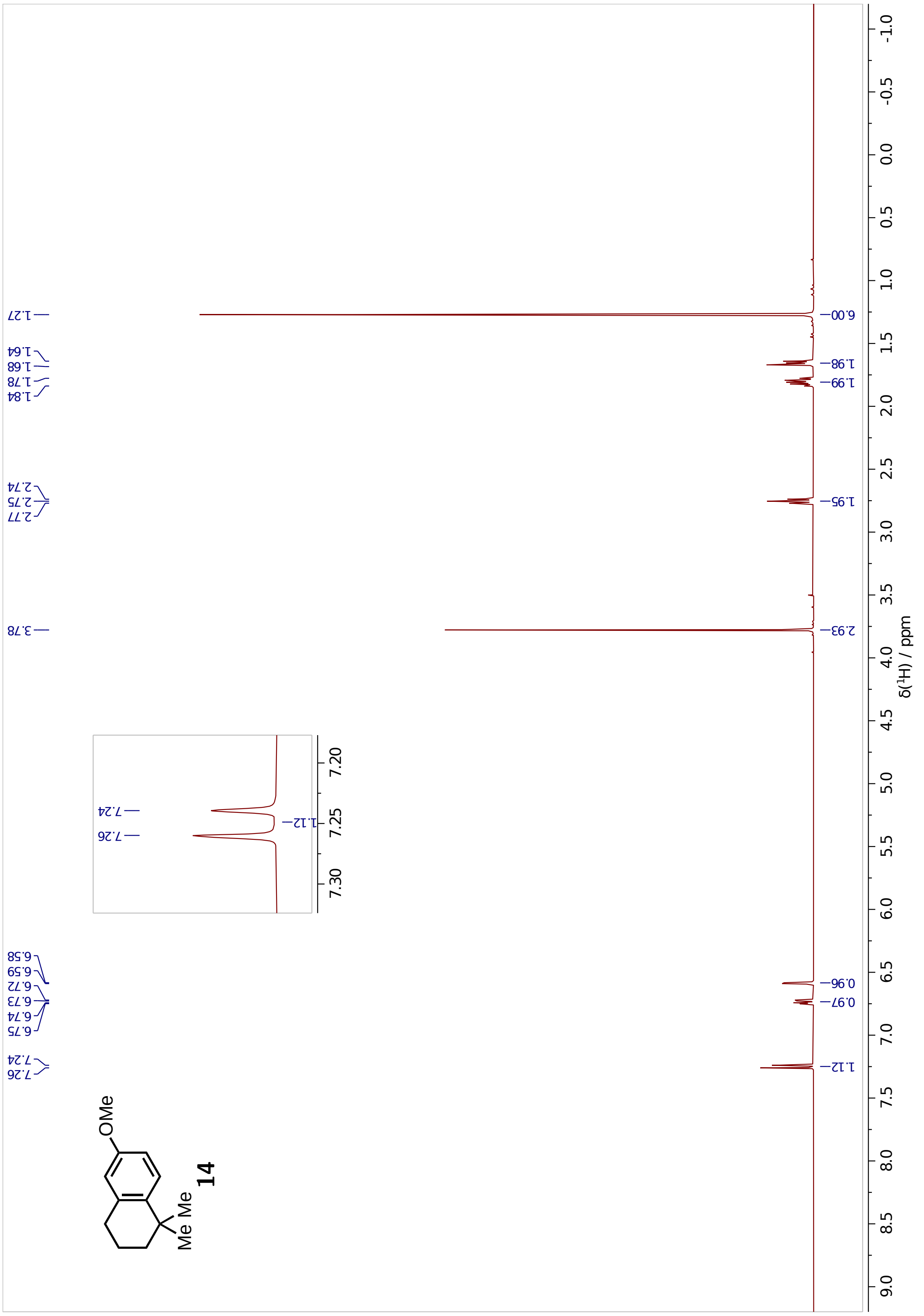


**Spectrum 7** *$^{1}$H-NMR spectrum of substance **14** measured in $CDCl_3$ at* 400 MHz.

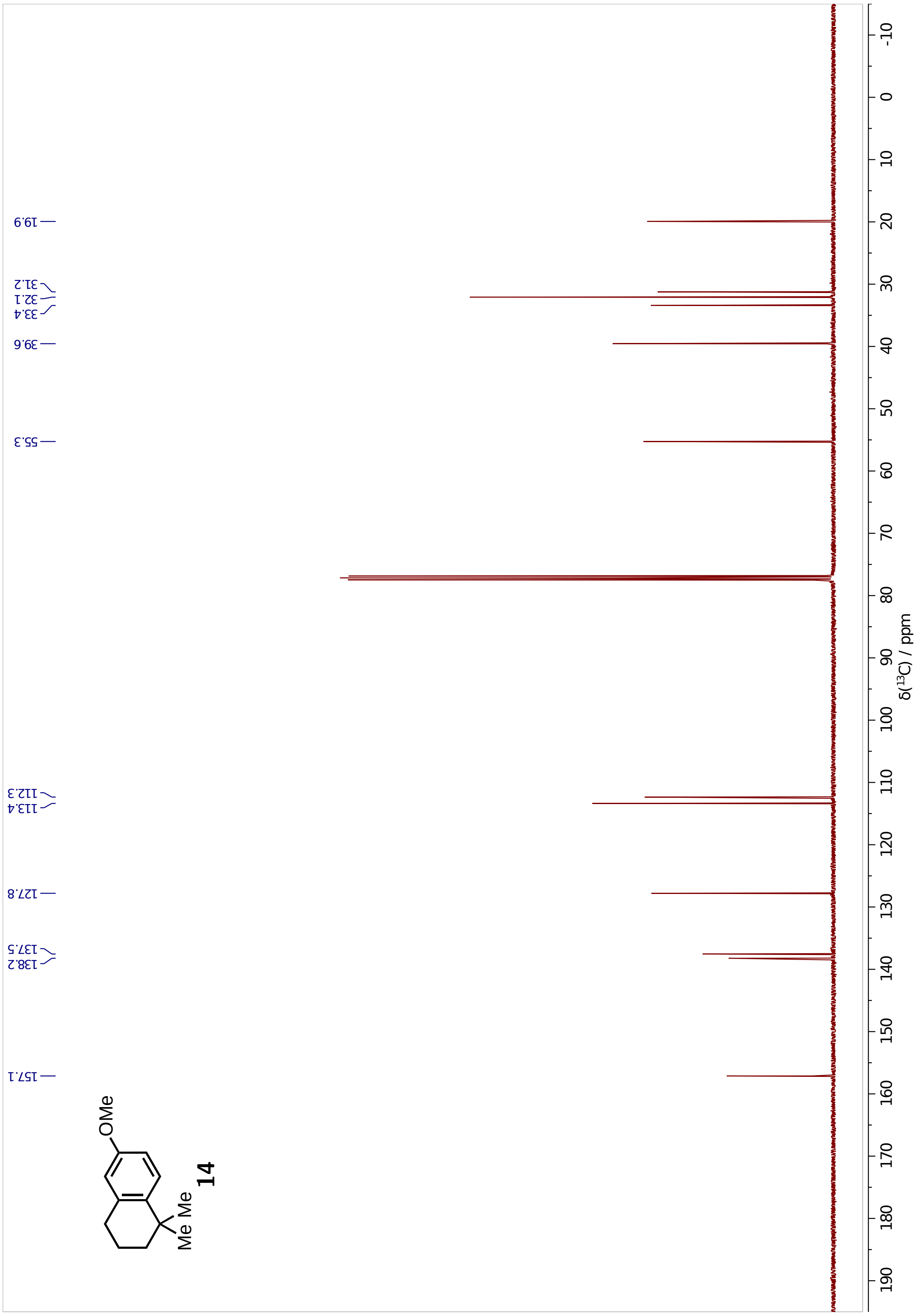


**Spectrum 8** *$^{13}$C-NMR spectrum of substance **14** measured in CDCl$_3$ at* 101 MHz.

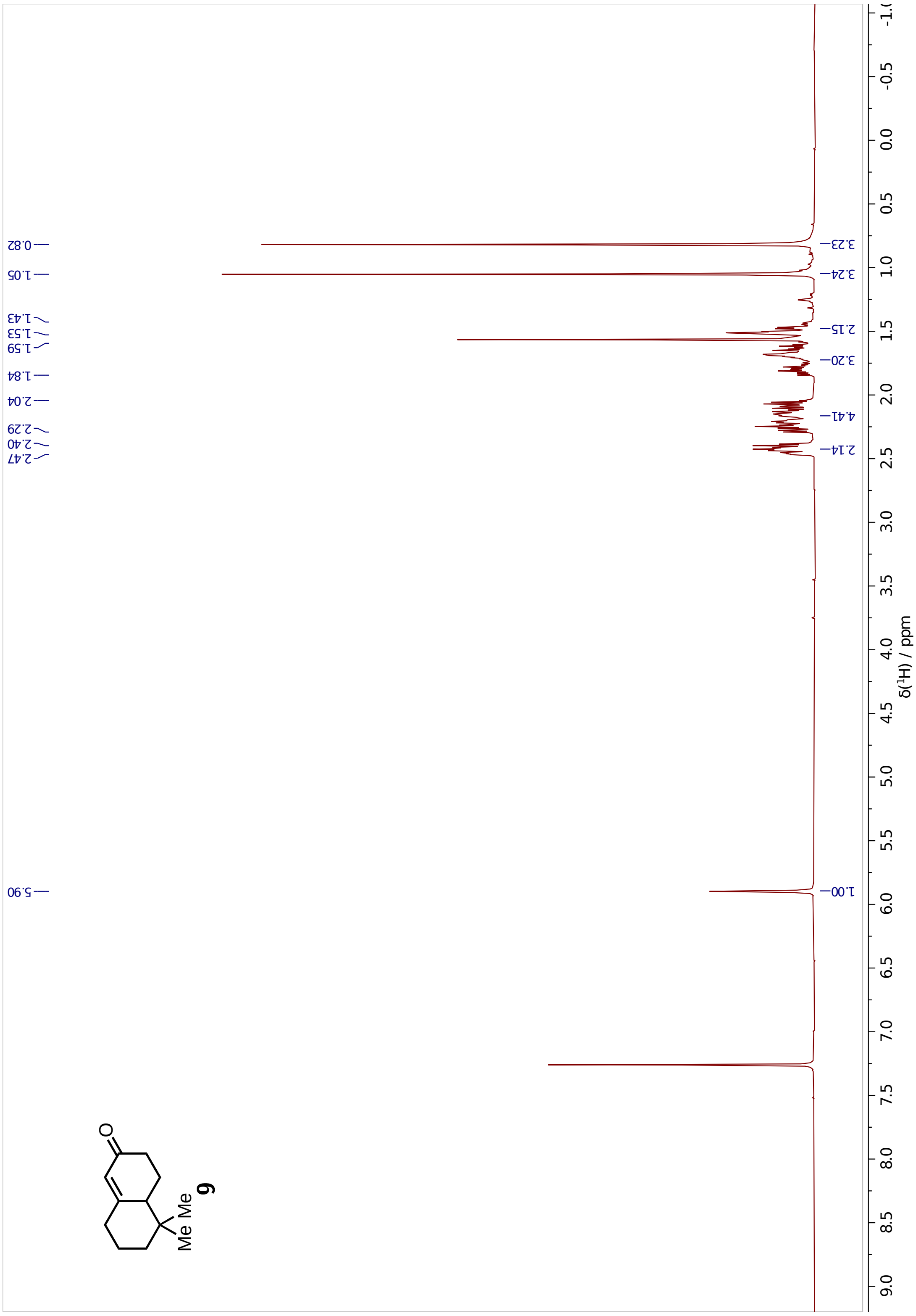


**Spectrum 9** *$^{1}$H-NMR spectrum of substance* ***9*** *measured in CDCl$_3$ at* 400 MHz.

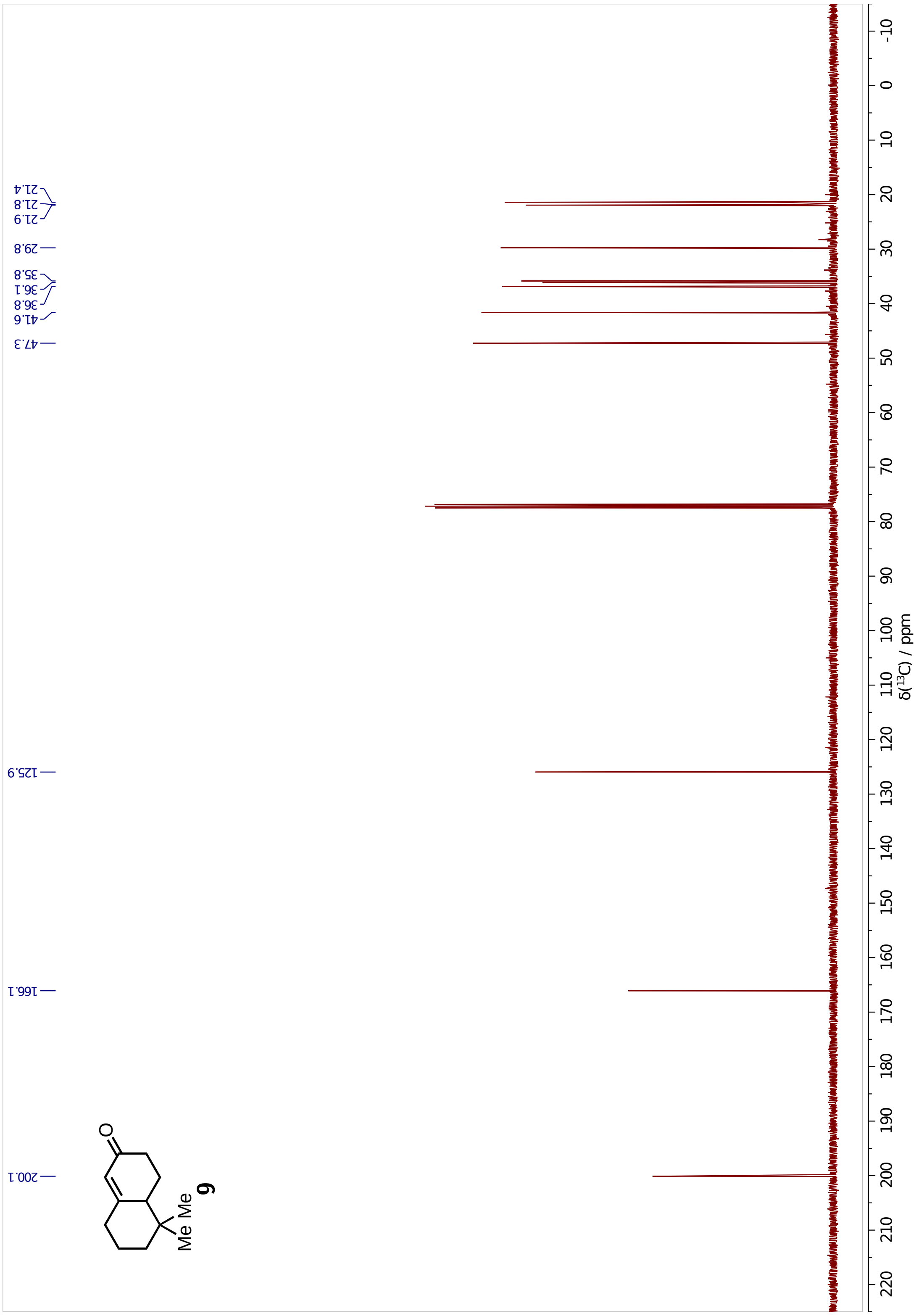


**Spectrum 10** *$^{13}$C-NMR spectrum of substance **9** measured in $CDCl_3$ at* 101 MHz.

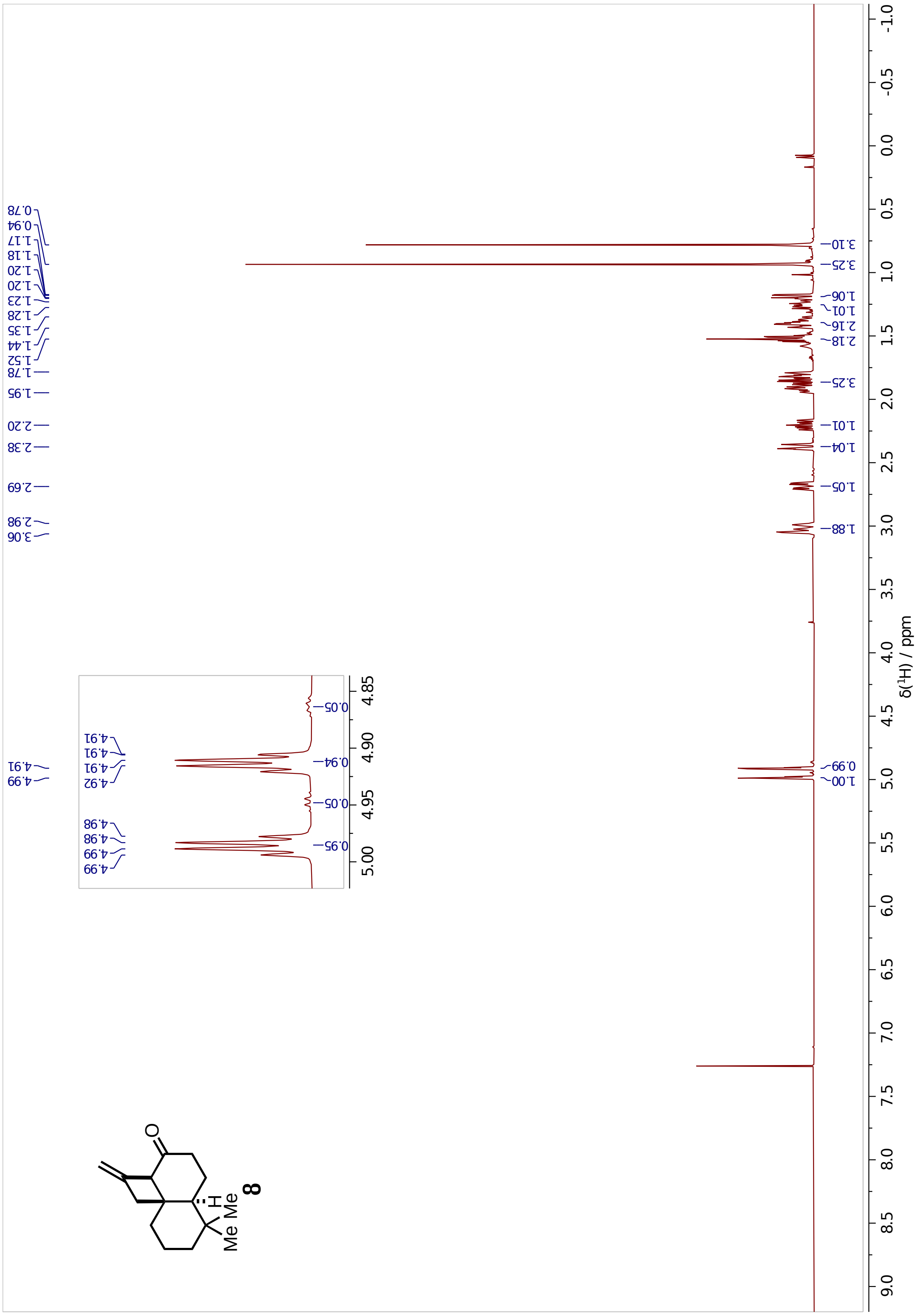


**Spectrum 11** *$^1$H-NMR spectrum of substance **8** measured in $CDCl_3$ at* 500 MHz.

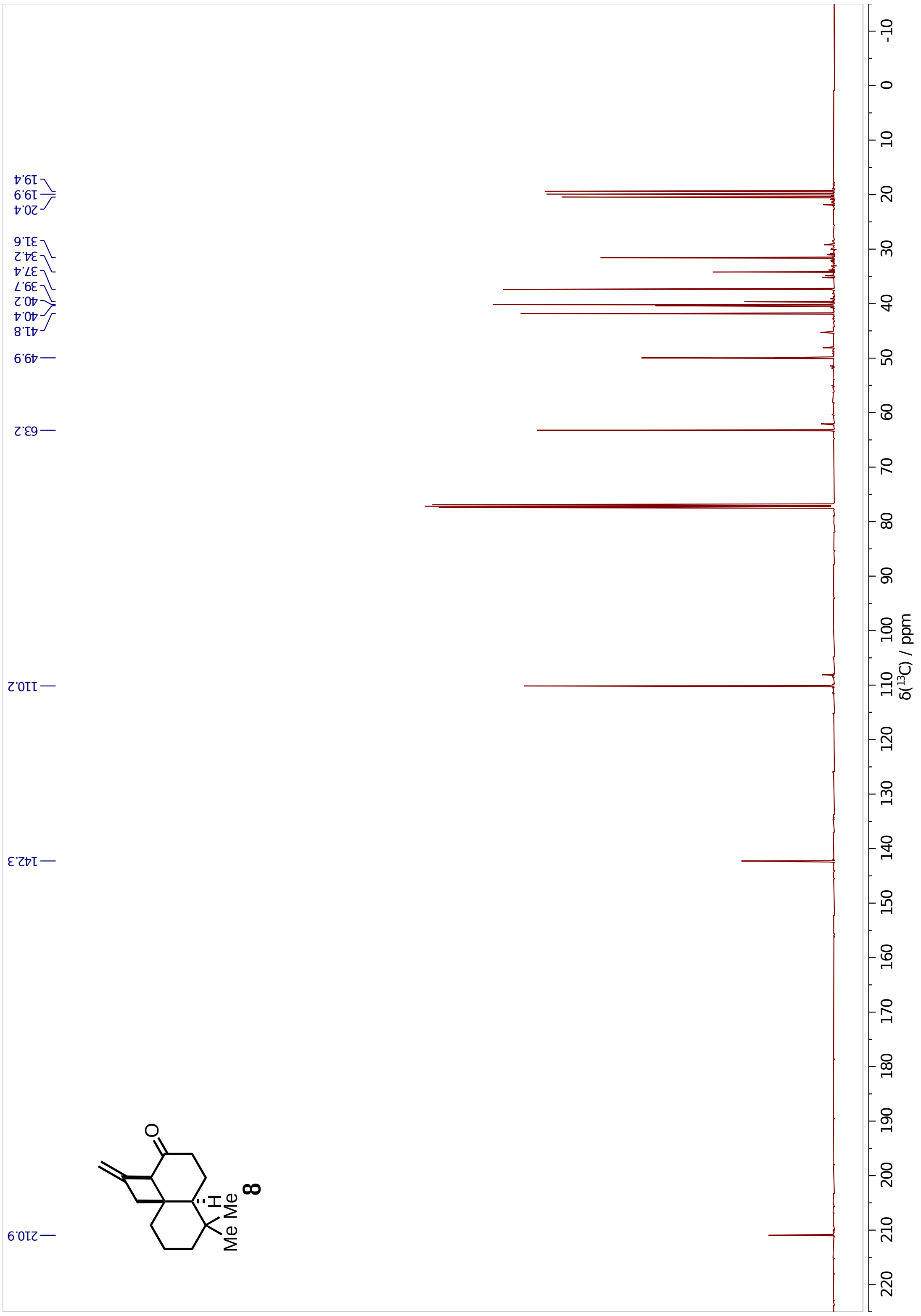


**Spectrum 12** *$^{13}$C-NMR spectrum of substance **8** measured in $CDCl_3$ at* 126 MHz.

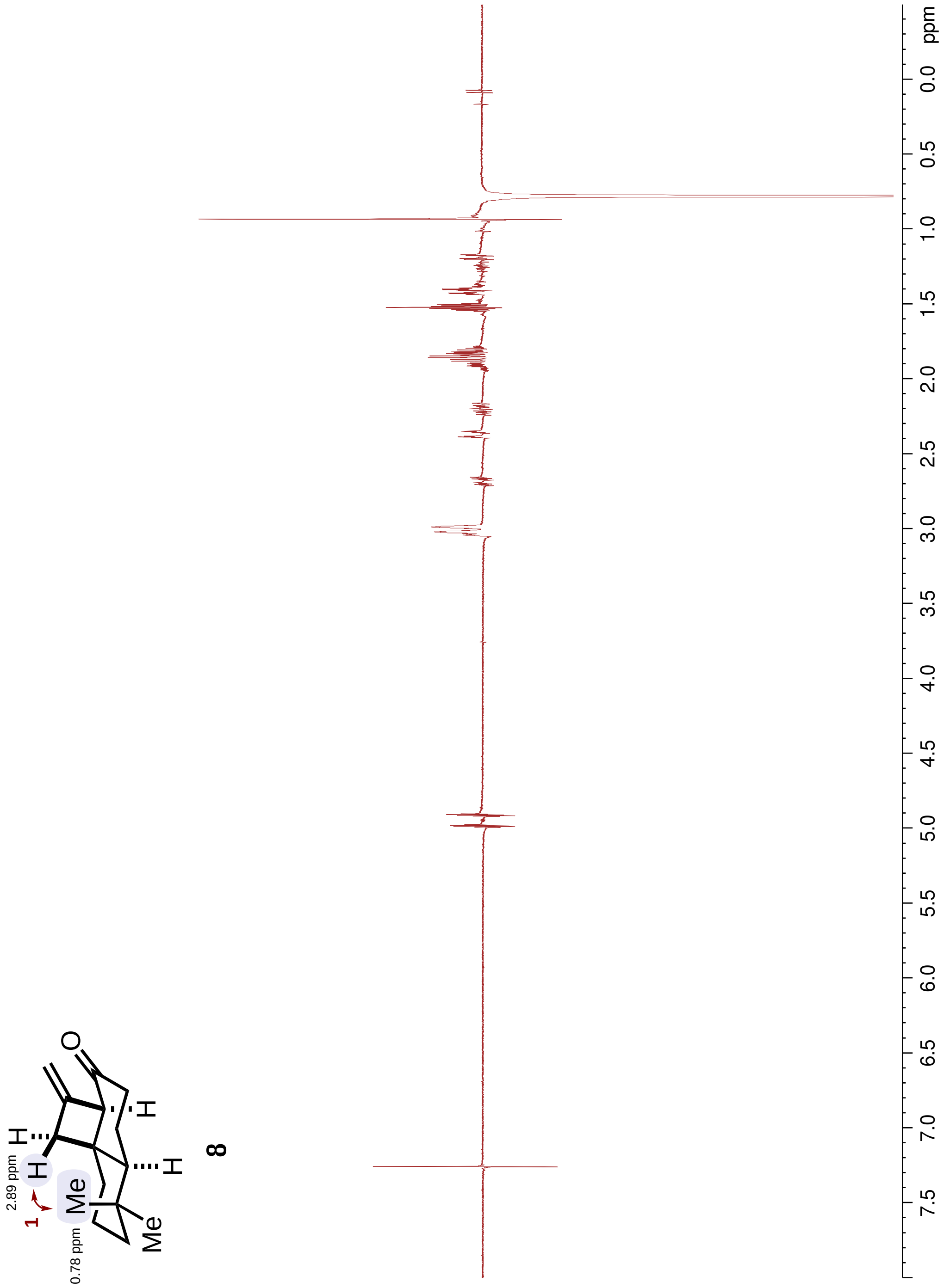


**Spectrum 13** *Nuclear Overhauser effects with excitation of the methyl group at* 0.78 ppm *measured in* $CDCl_3$ *at* 500 MHz.

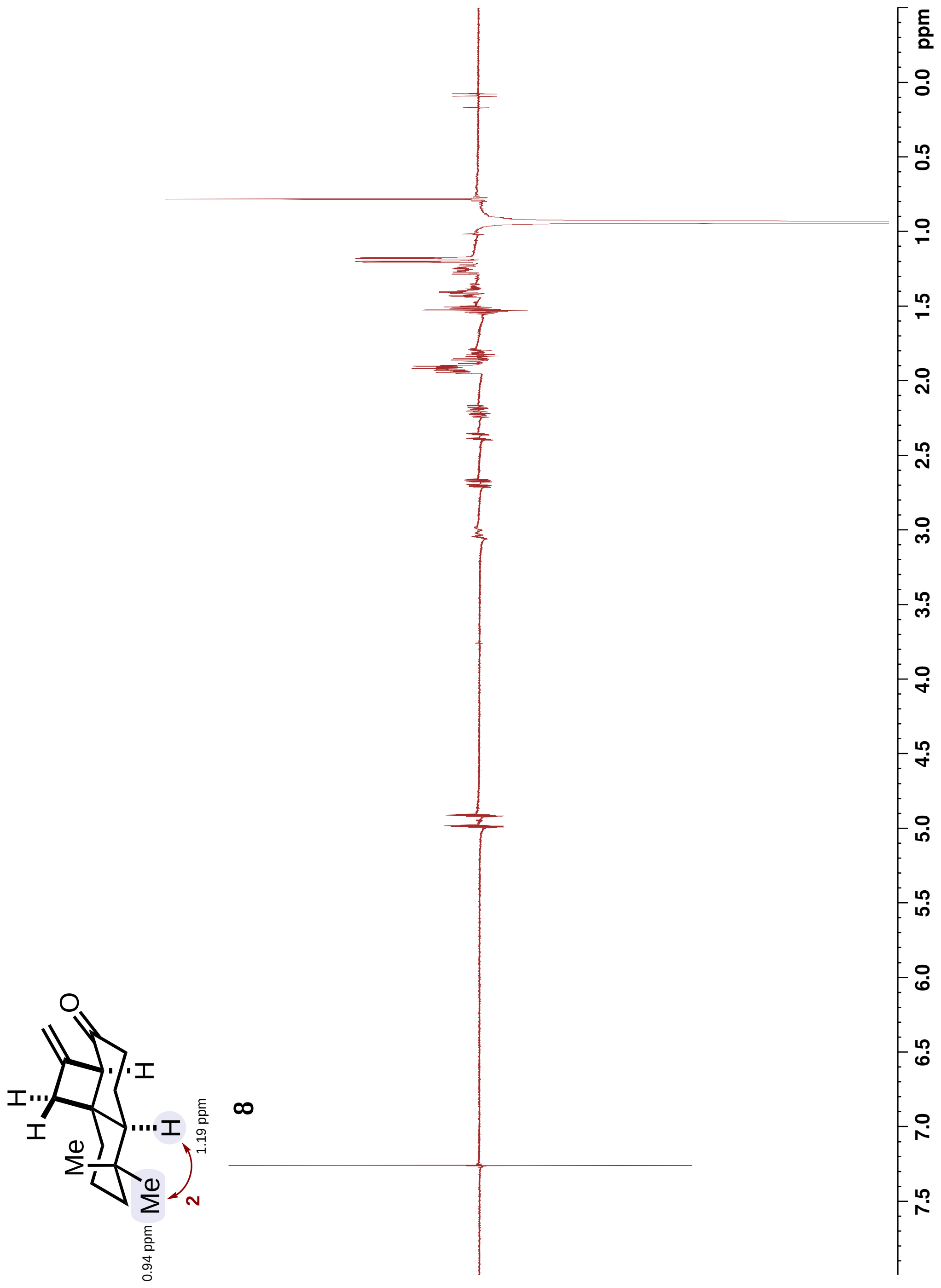


**Spectrum 14** *Nuclear Overhauser effects with excitation of the methyl group at* 0.94 ppm *measured in* $CDCl_3$ *at* 500 MHz.

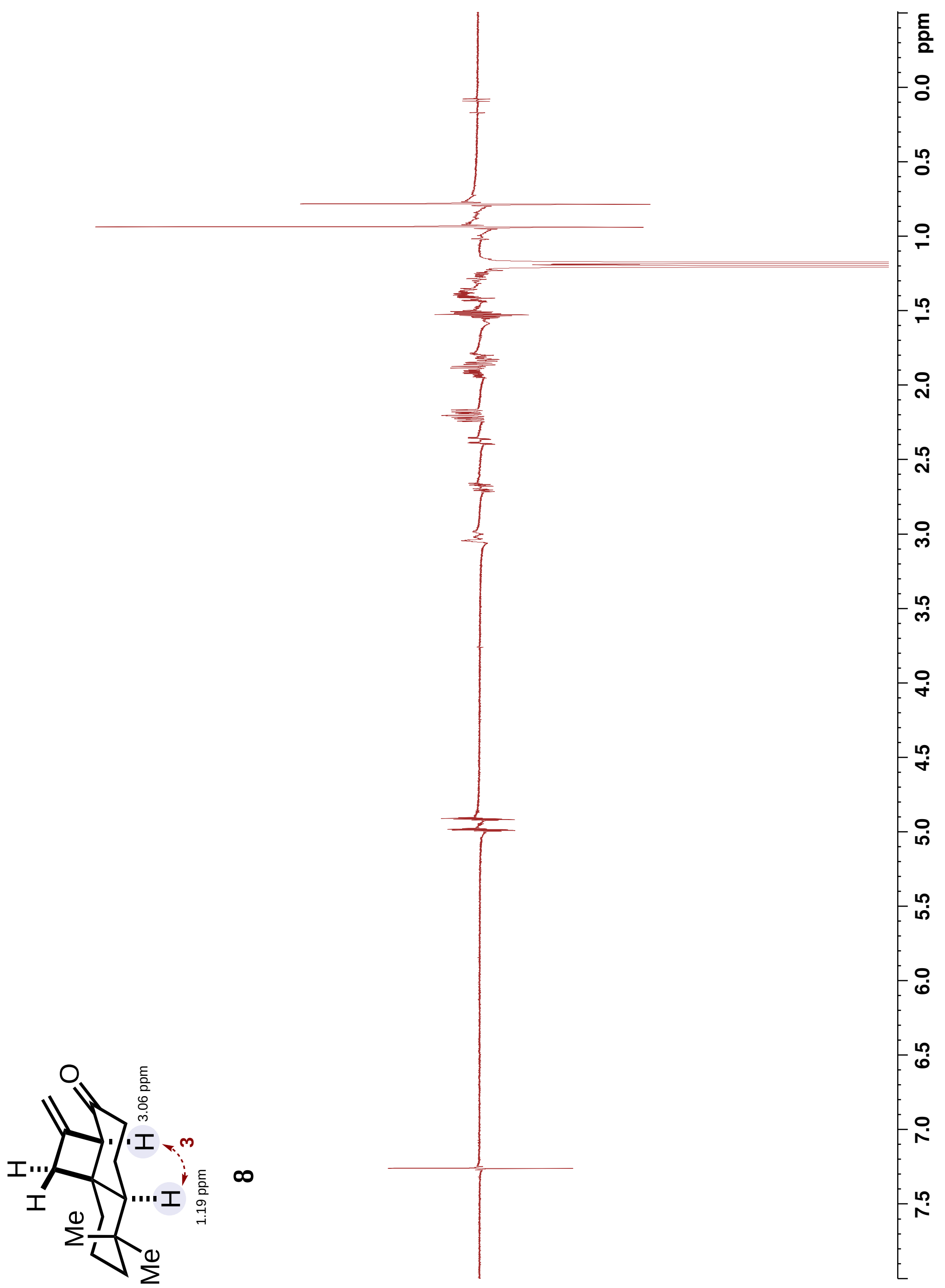


**Spectrum 15** *Nuclear Overhauser effects with excitation of the proton at* 1.19 ppm *measured in* $CDCl_3$ *at* 500 MHz.

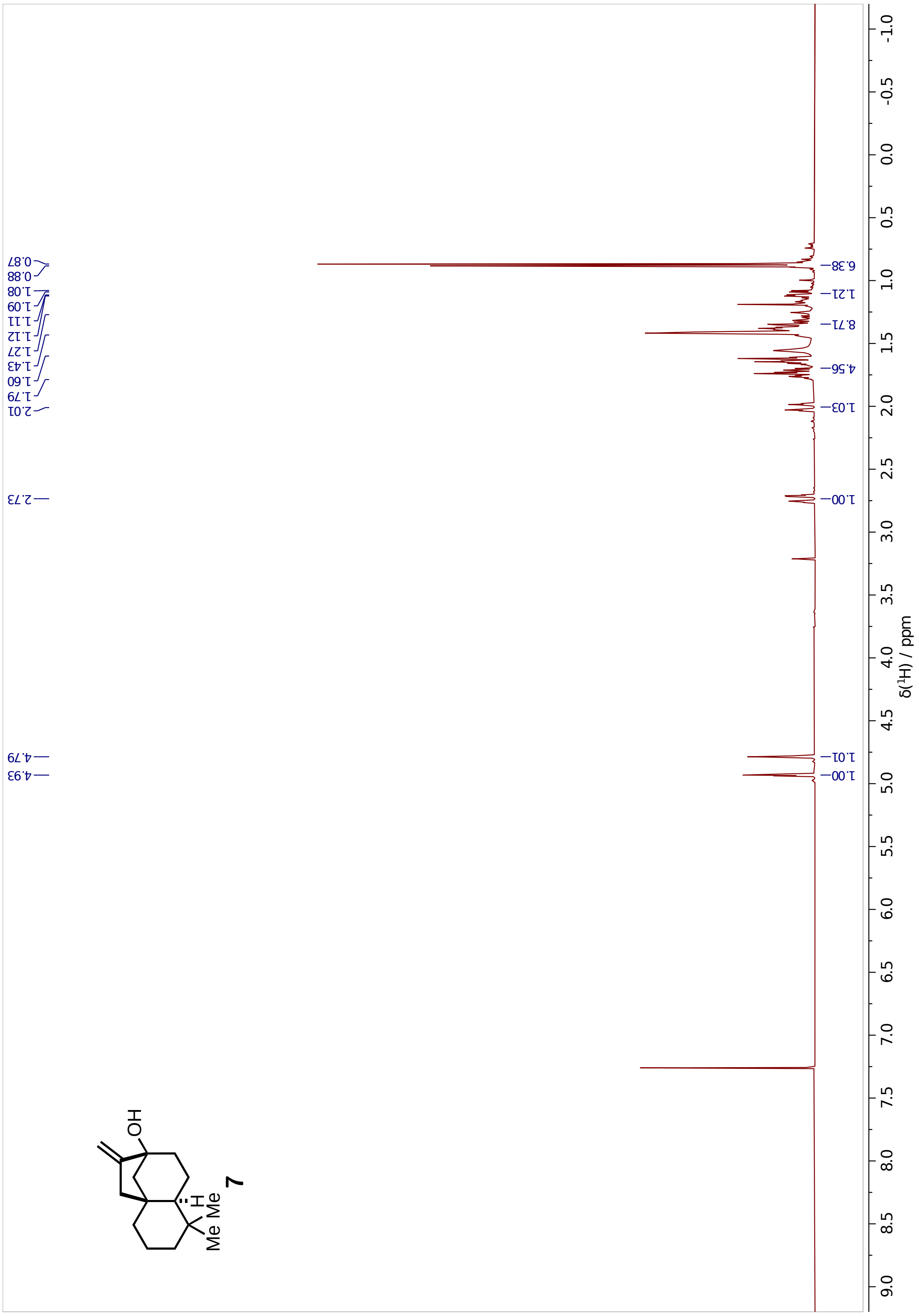


**Spectrum 16** *$^{1}$H-NMR spectrum of substance **7** measured in $CDCl_3$ at* 400 MHz.

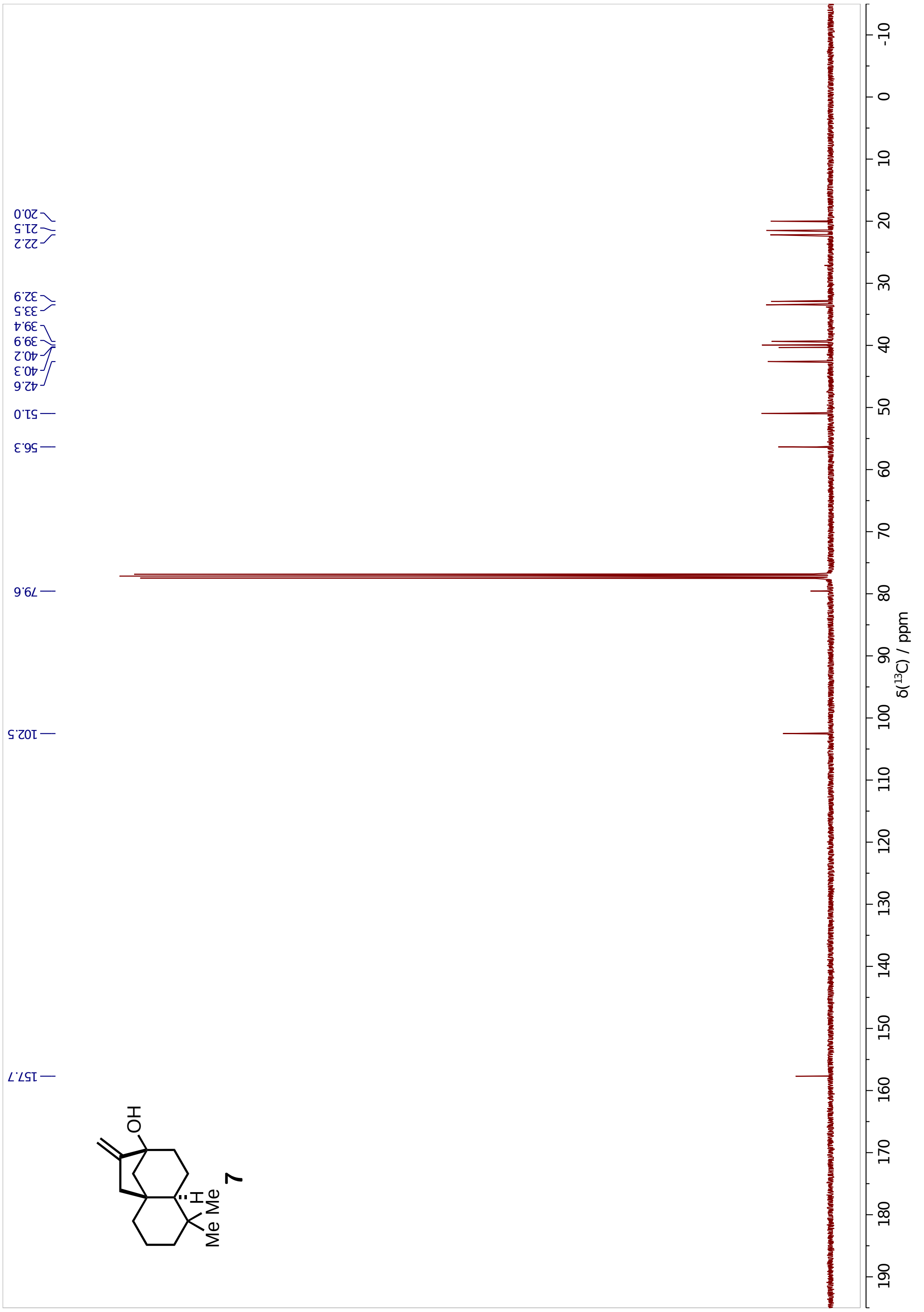


**Spectrum 17** *$^{13}$C-NMR spectrum of substance* ***7*** *measured in $CDCl_3$ at* 101 MHz.

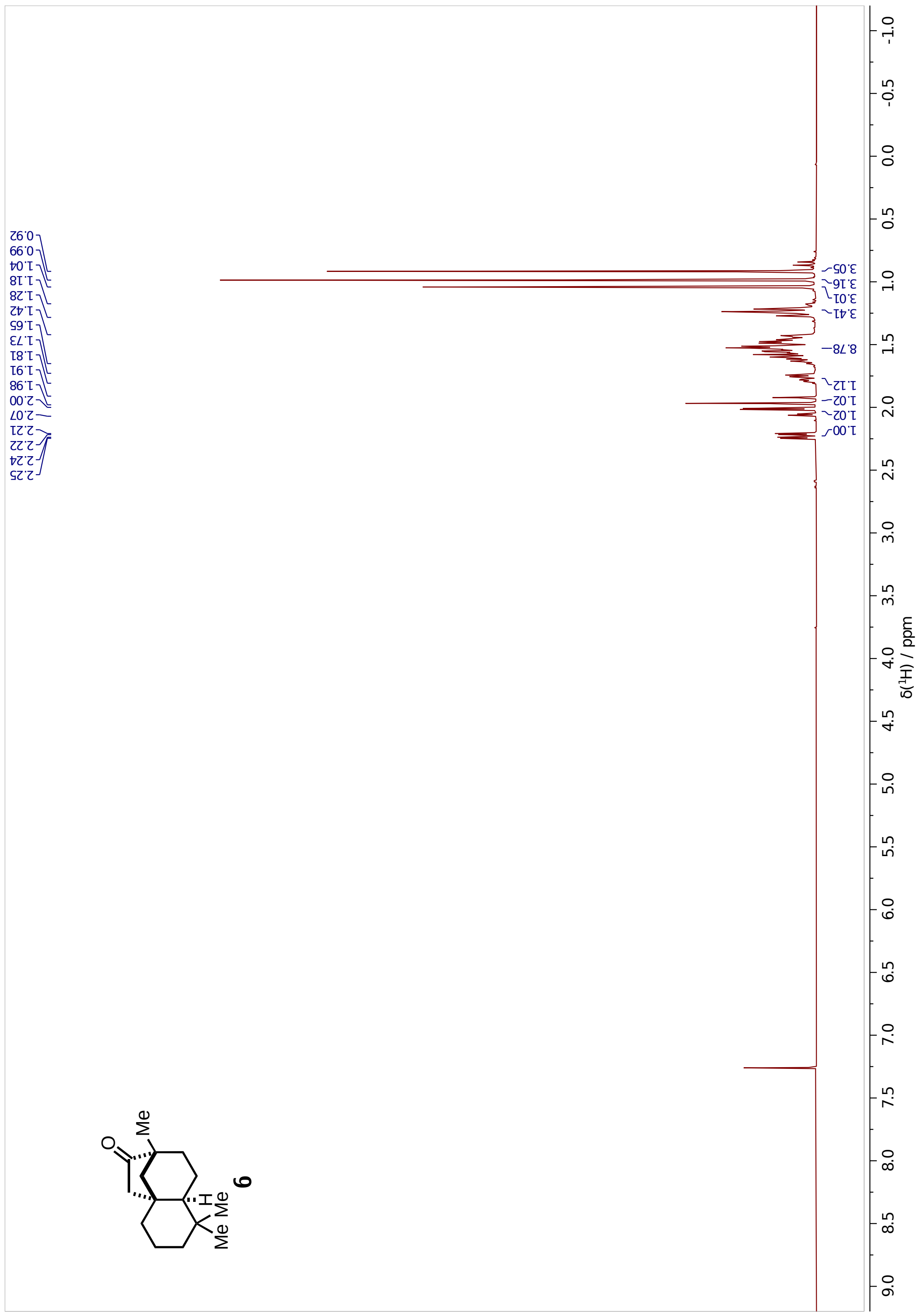


**Spectrum 18** *$^{1}$H-NMR spectrum of substance* ***6*** *measured in* $CDCl_3$ *at* 400 MHz.

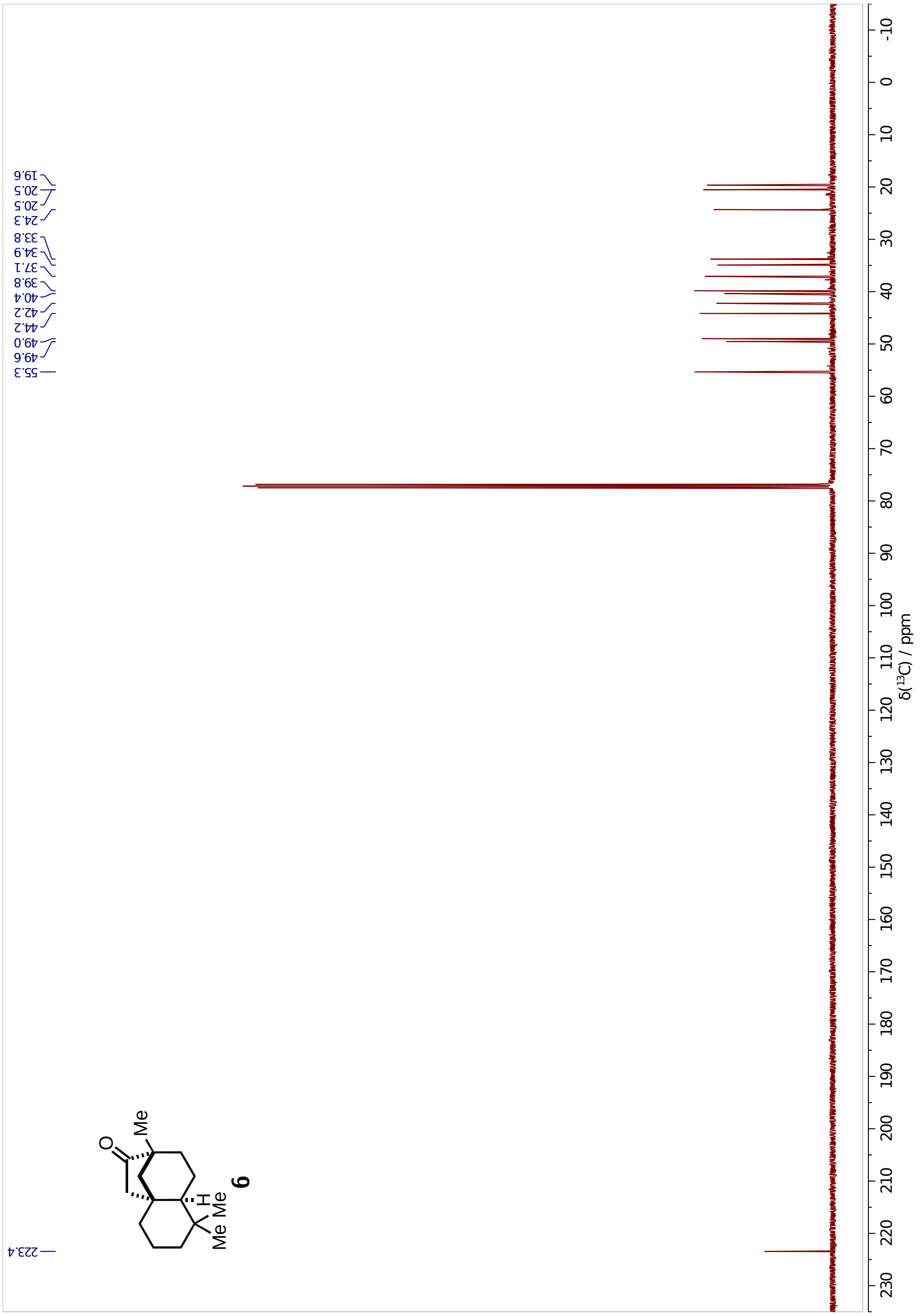


**Spectrum 19** *$^{13}$C-NMR spectrum of substance **6** measured in $CDCl_3$ at* 101 MHz.

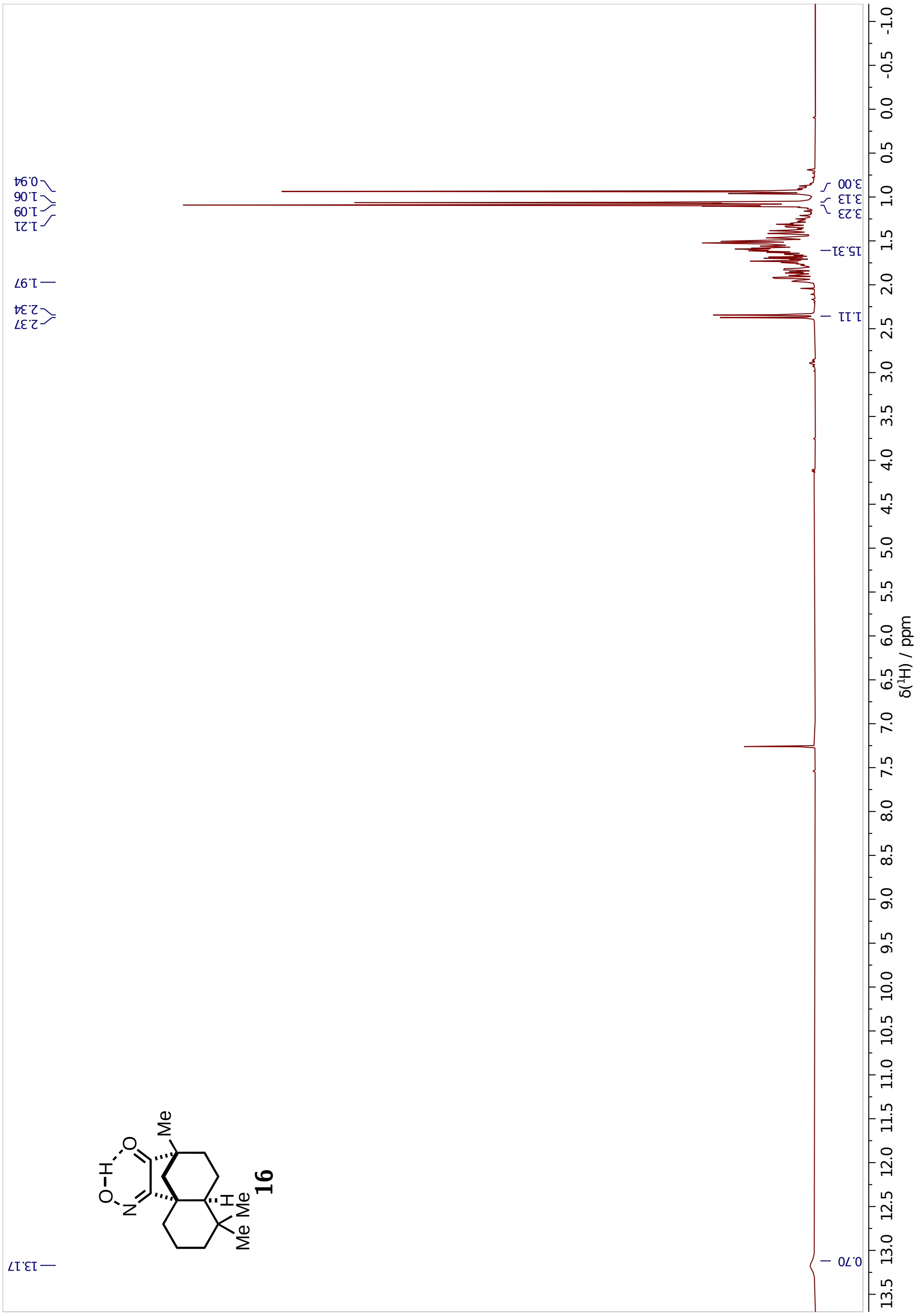


**Spectrum 20** *$^{1}$H-NMR spectrum of substance* ***16*** *measured in $CDCl_3$ at* 400 MHz.

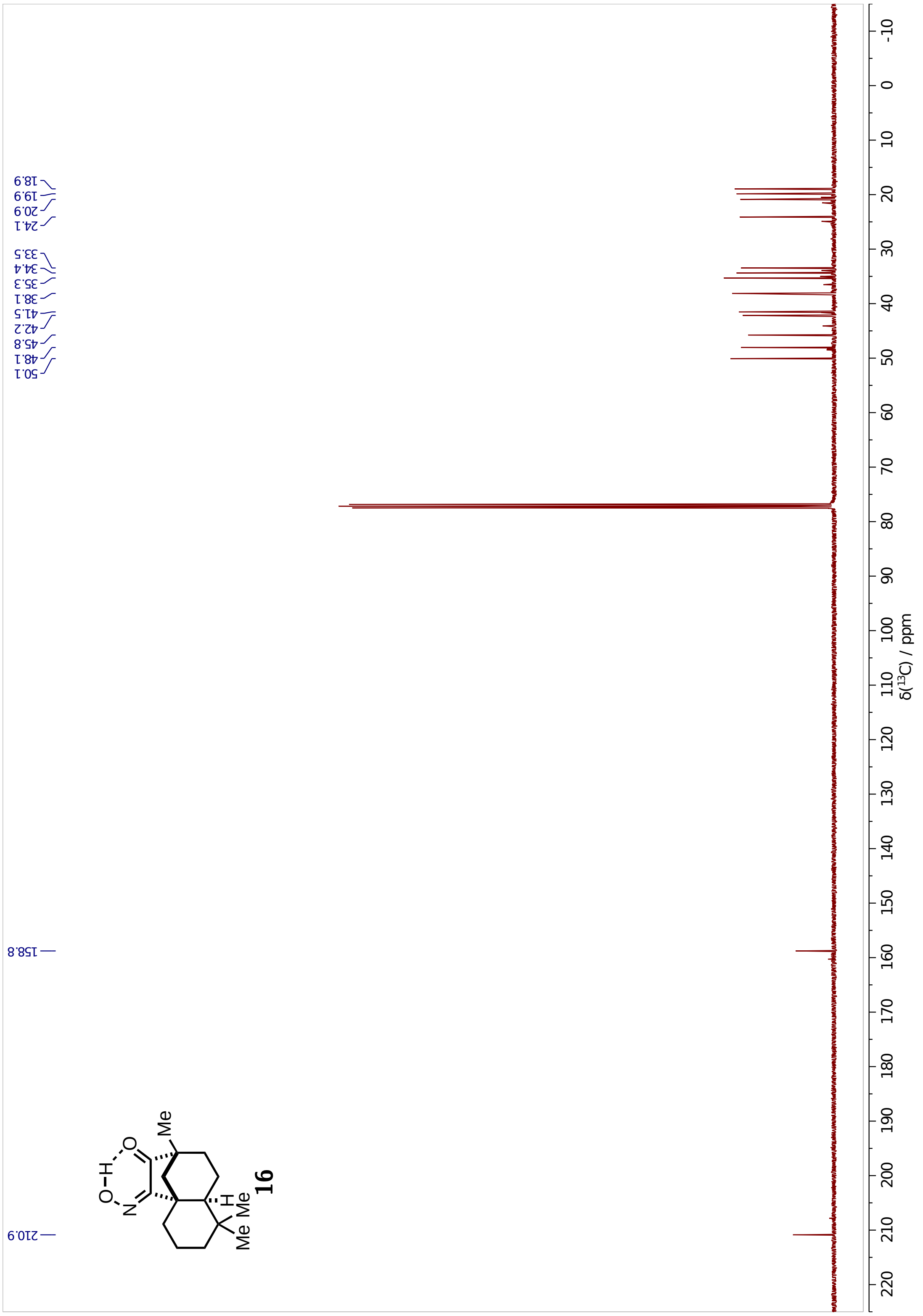


**Spectrum 21** *$^{13}$C-NMR spectrum of substance **16** measured in $CDCl_3$ at* 101 MHz.

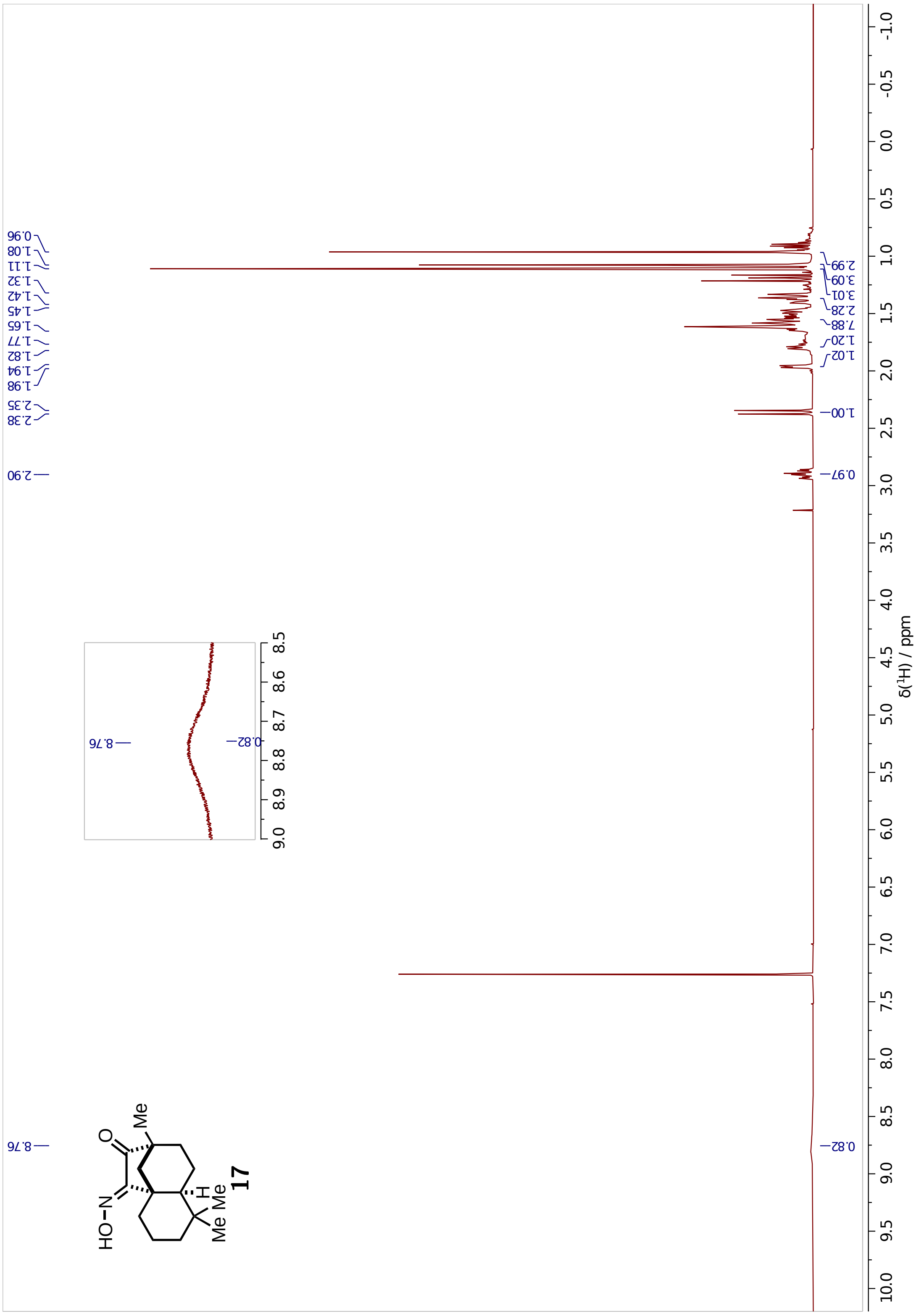


**Spectrum 22** *$^{1}$H-NMR spectrum of substance* ***17*** *measured in $CDCl_3$ at* 400 MHz.

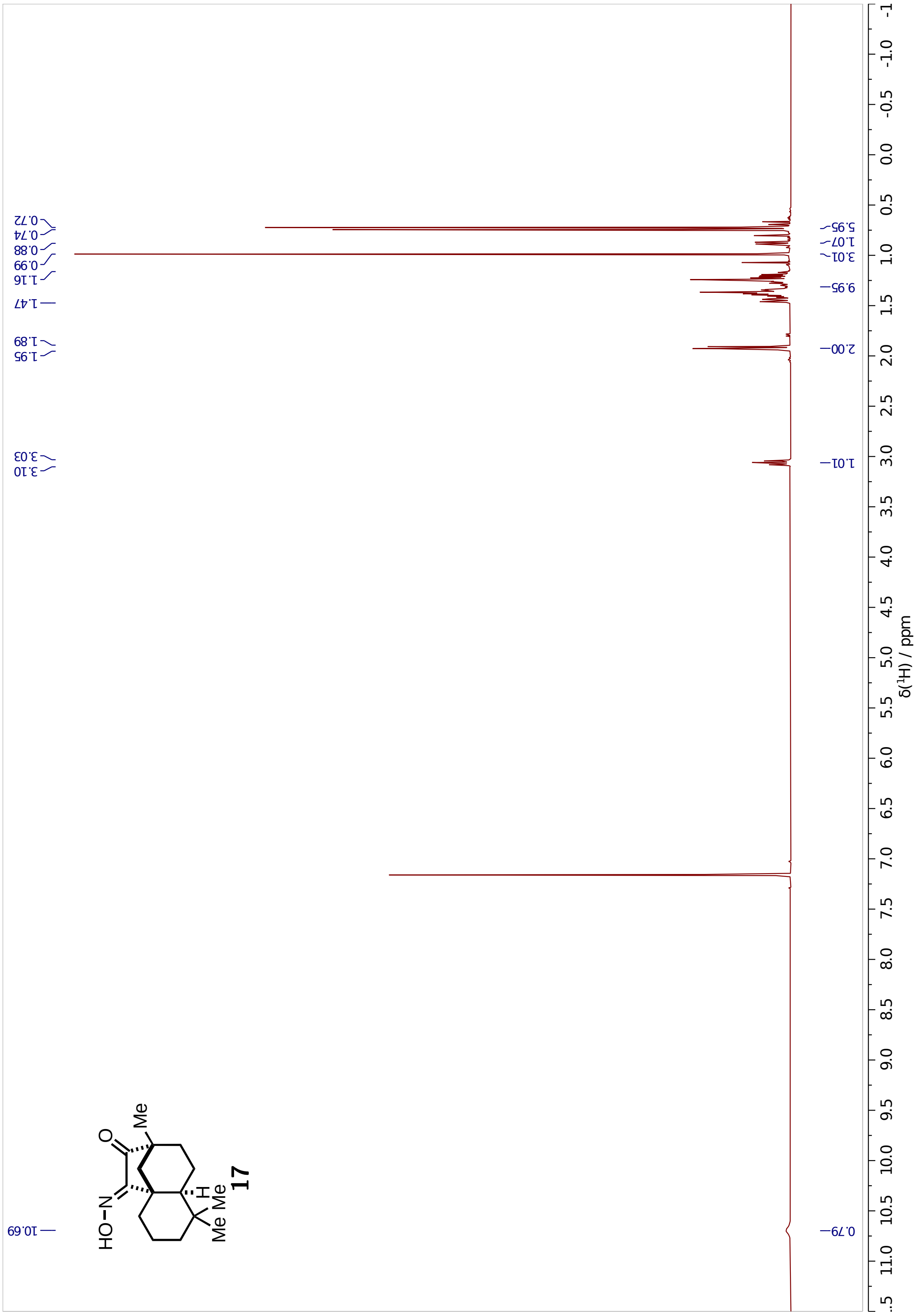


**Spectrum 23** *1H-NMR spectrum of substance **17** measured in $C_6D_6$ at* 600 MHz.

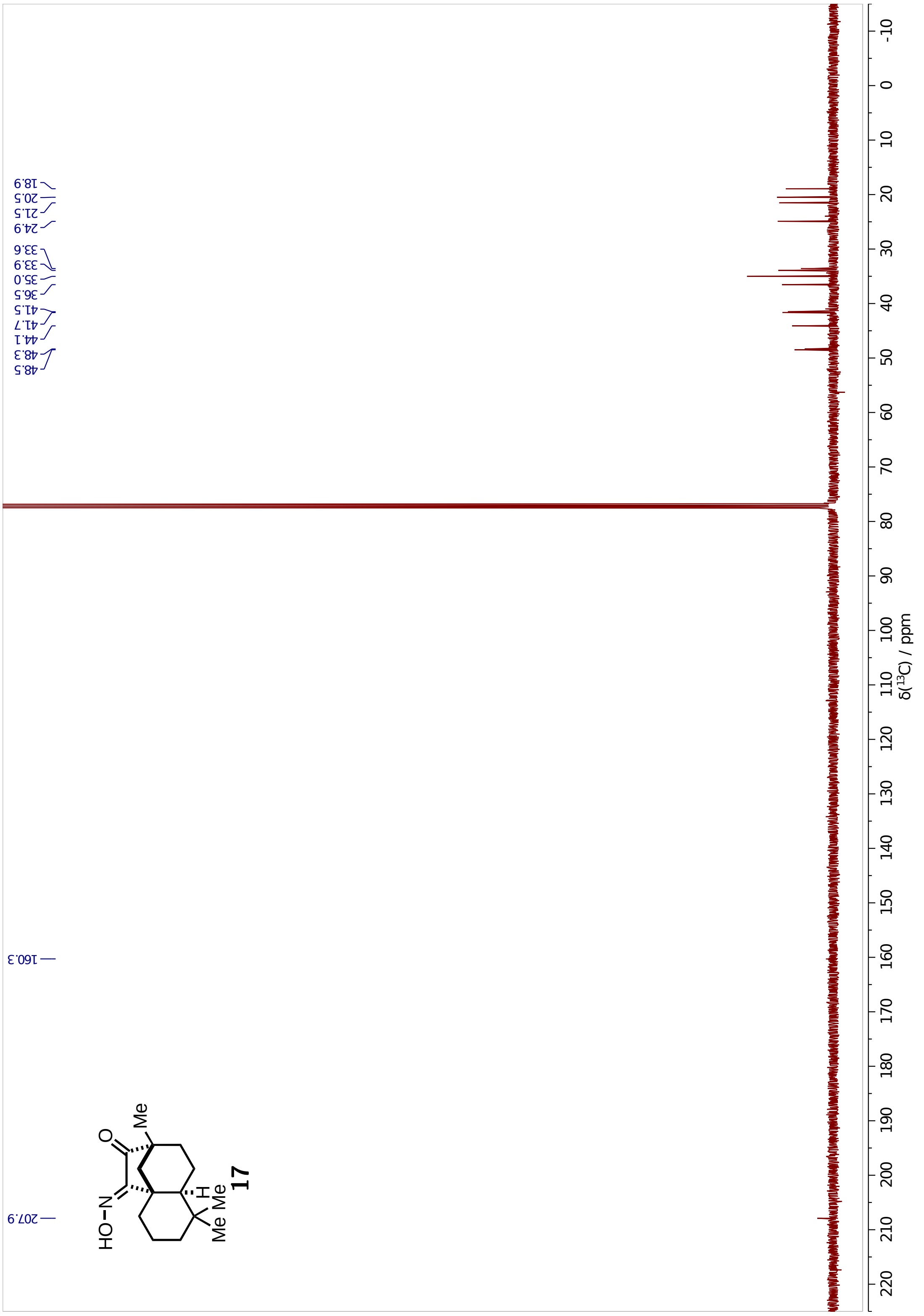


**Spectrum 24** *$^{13}$C-NMR spectrum of substance* ***17*** *measured in $CDCl_3$ at* 101 MHz.

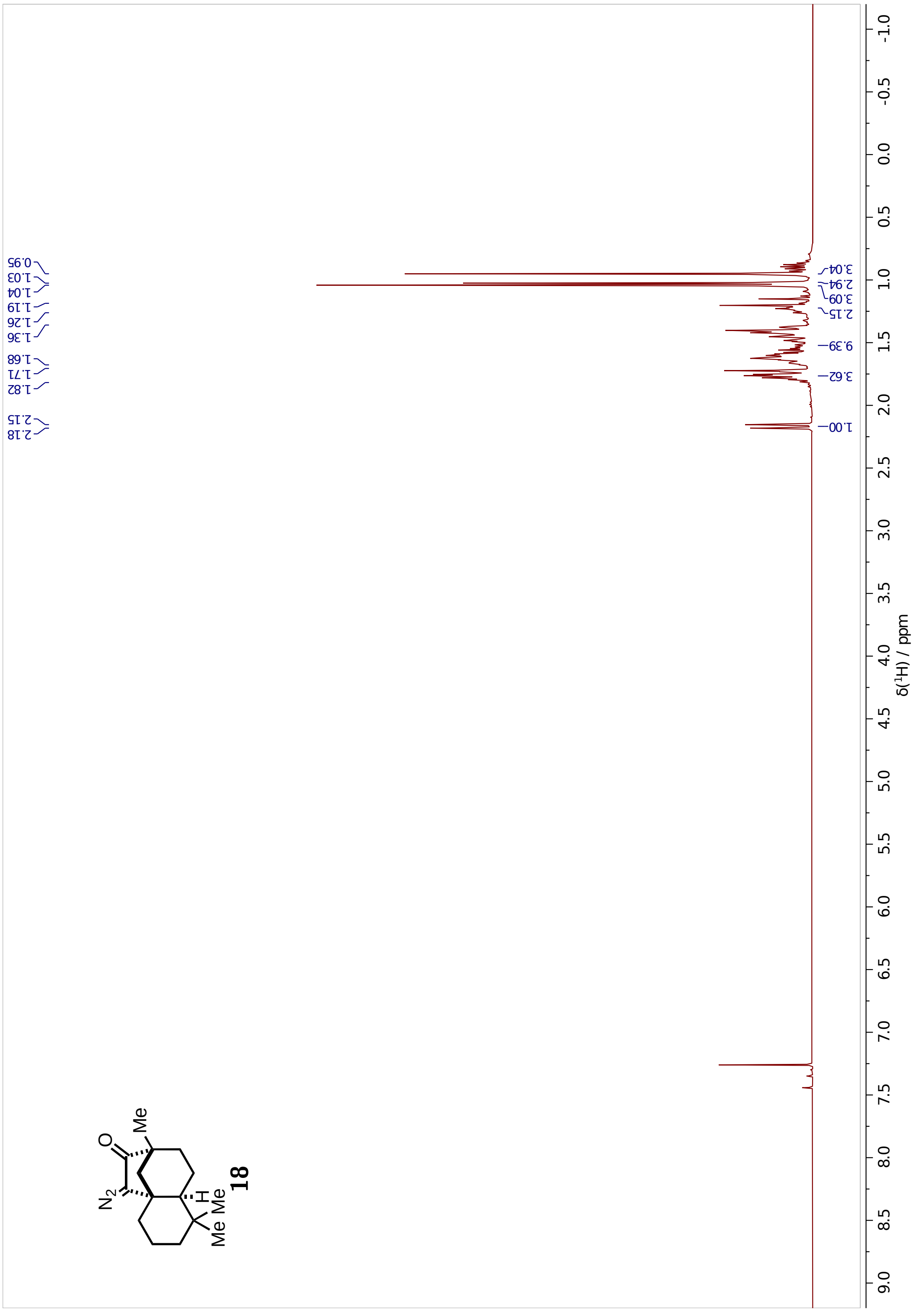


**Spectrum 25** *$^1$H-NMR spectrum of substance **18** measured in $CDCl_3$ at* 400 MHz *at 0 °C.*

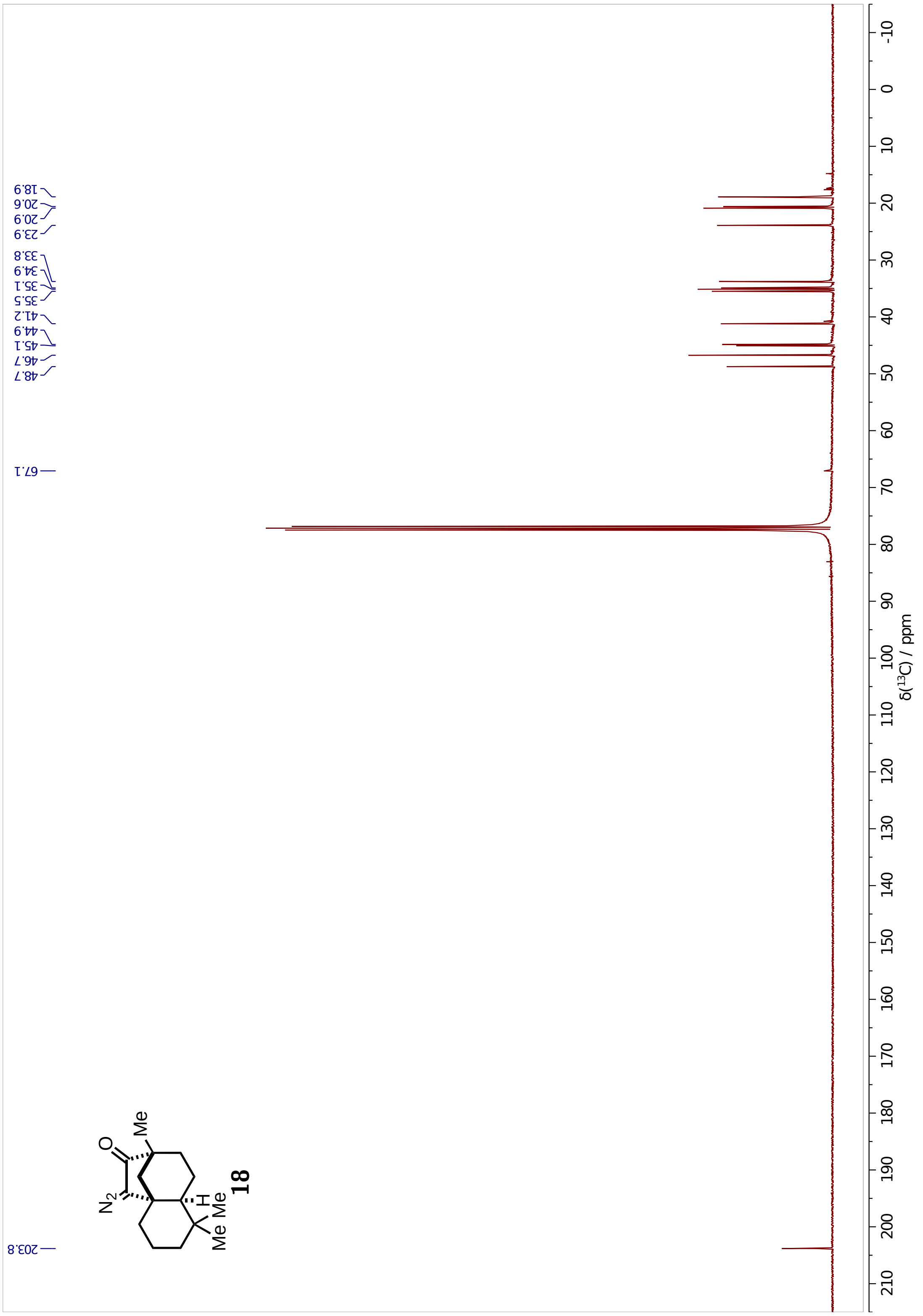


**Spectrum 26** *$^{13}$C-NMR spectrum of substance* ***18*** *measured in $CDCl_3$ at* 101 MHz *at 0 °C.*

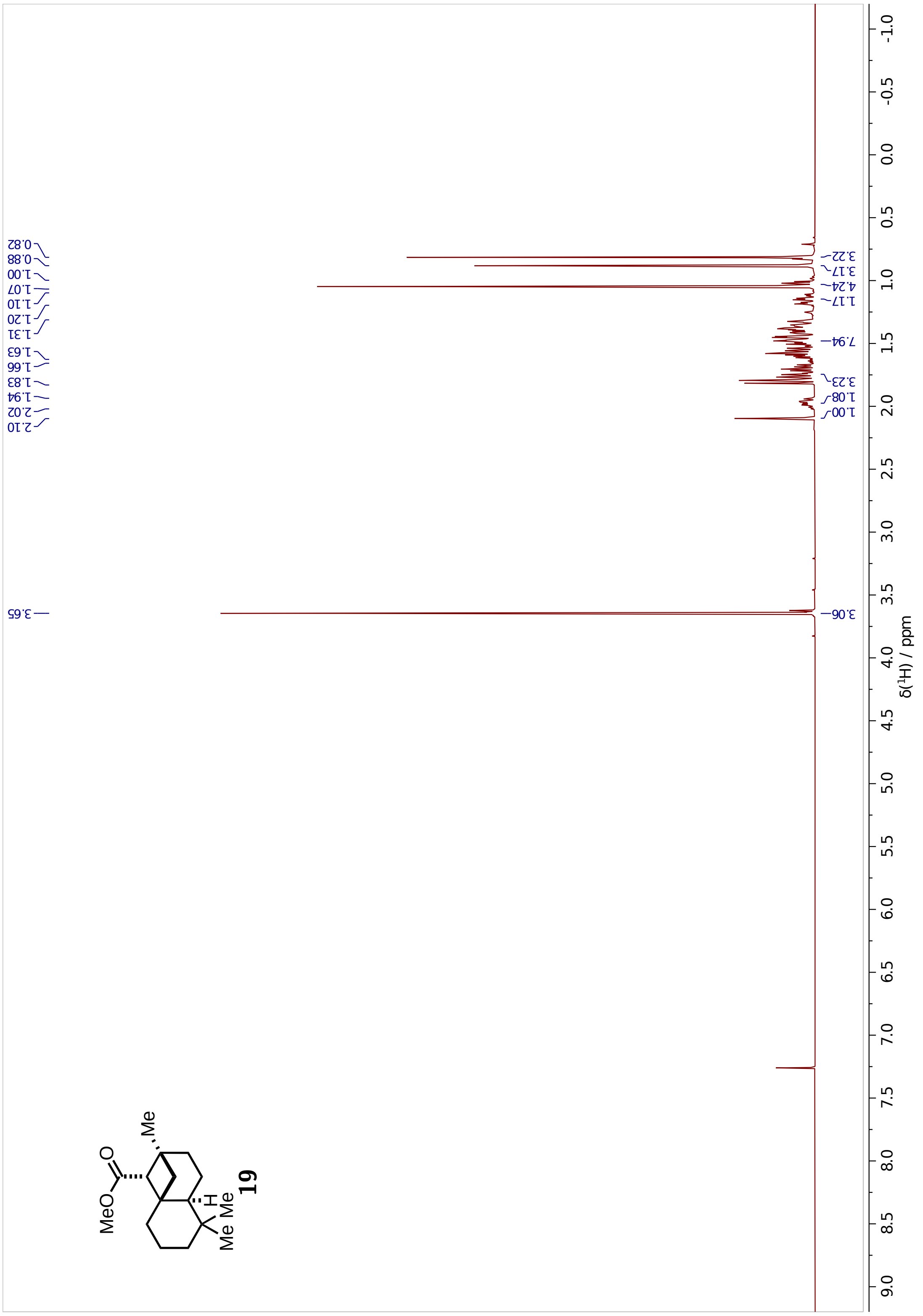


**Spectrum 27** *$^{1}$H-NMR spectrum of substance **19** measured in $CDCl_3$ at* 400 MHz.

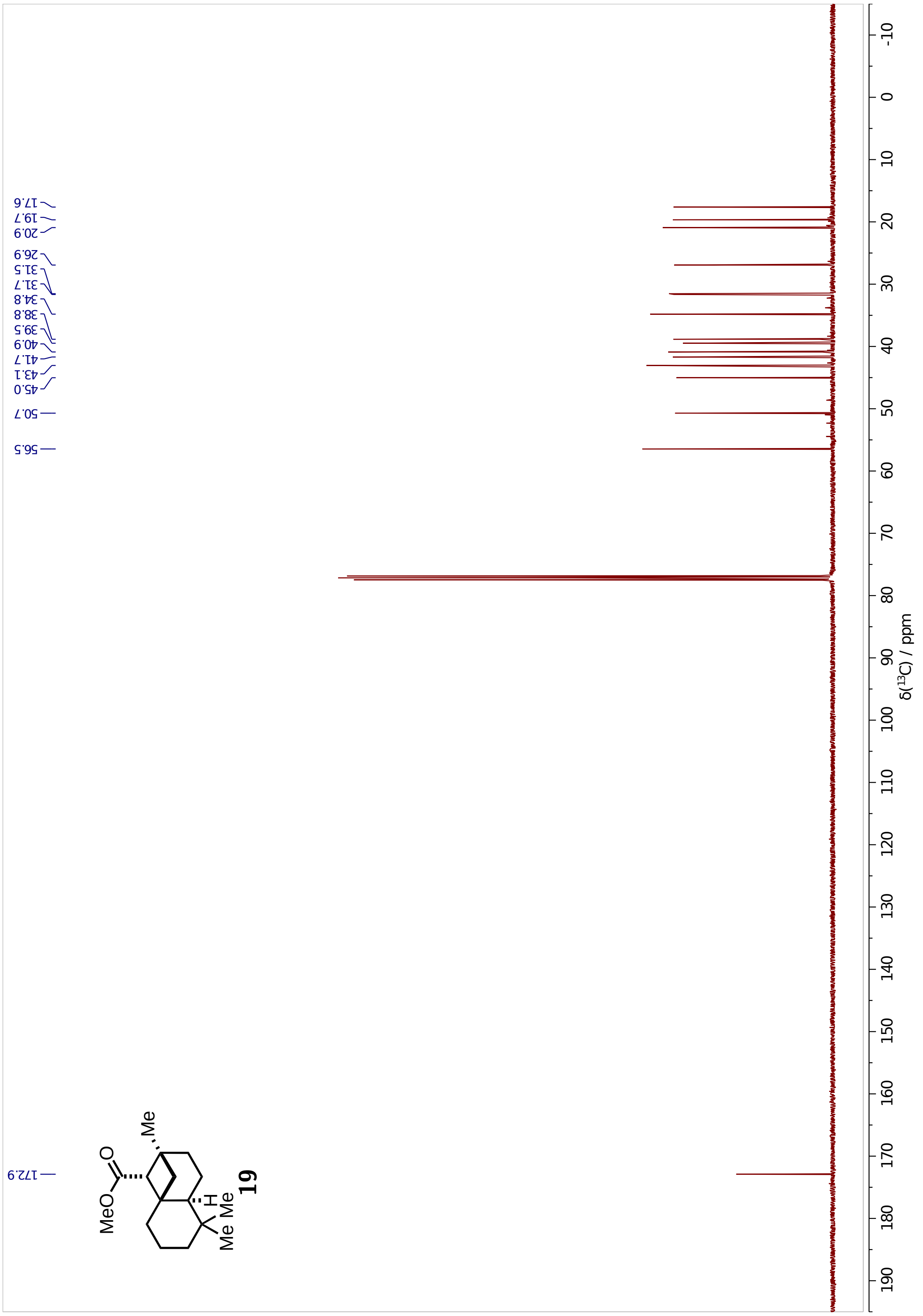


**Spectrum 28** *$^{13}$C-NMR spectrum of substance **19** measured in $CDCl_3$ at* 101 MHz.

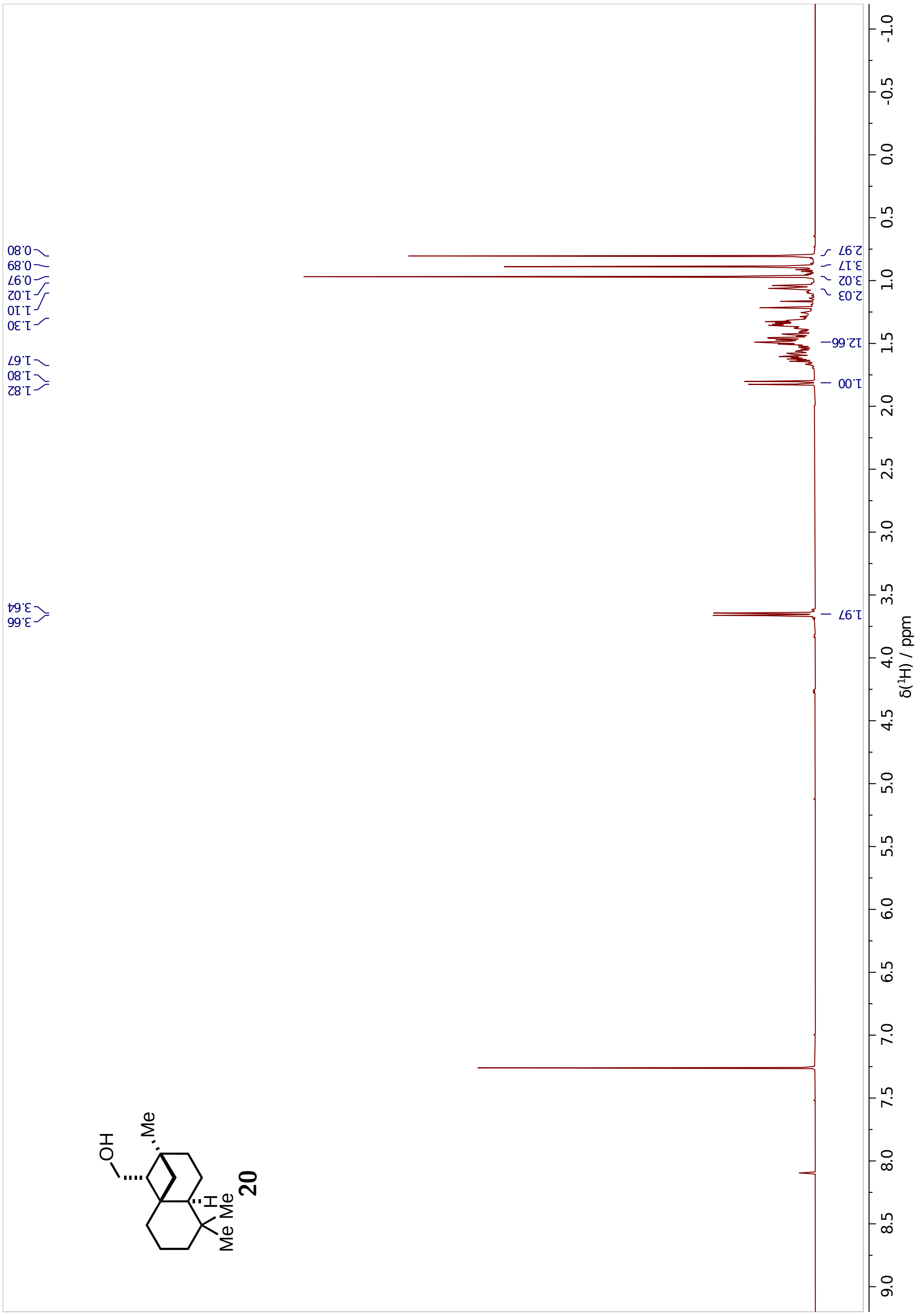


**Spectrum 29** *$^{1}$H-NMR spectrum of substance* ***20*** *measured in $CDCl_3$ at* 400 MHz.

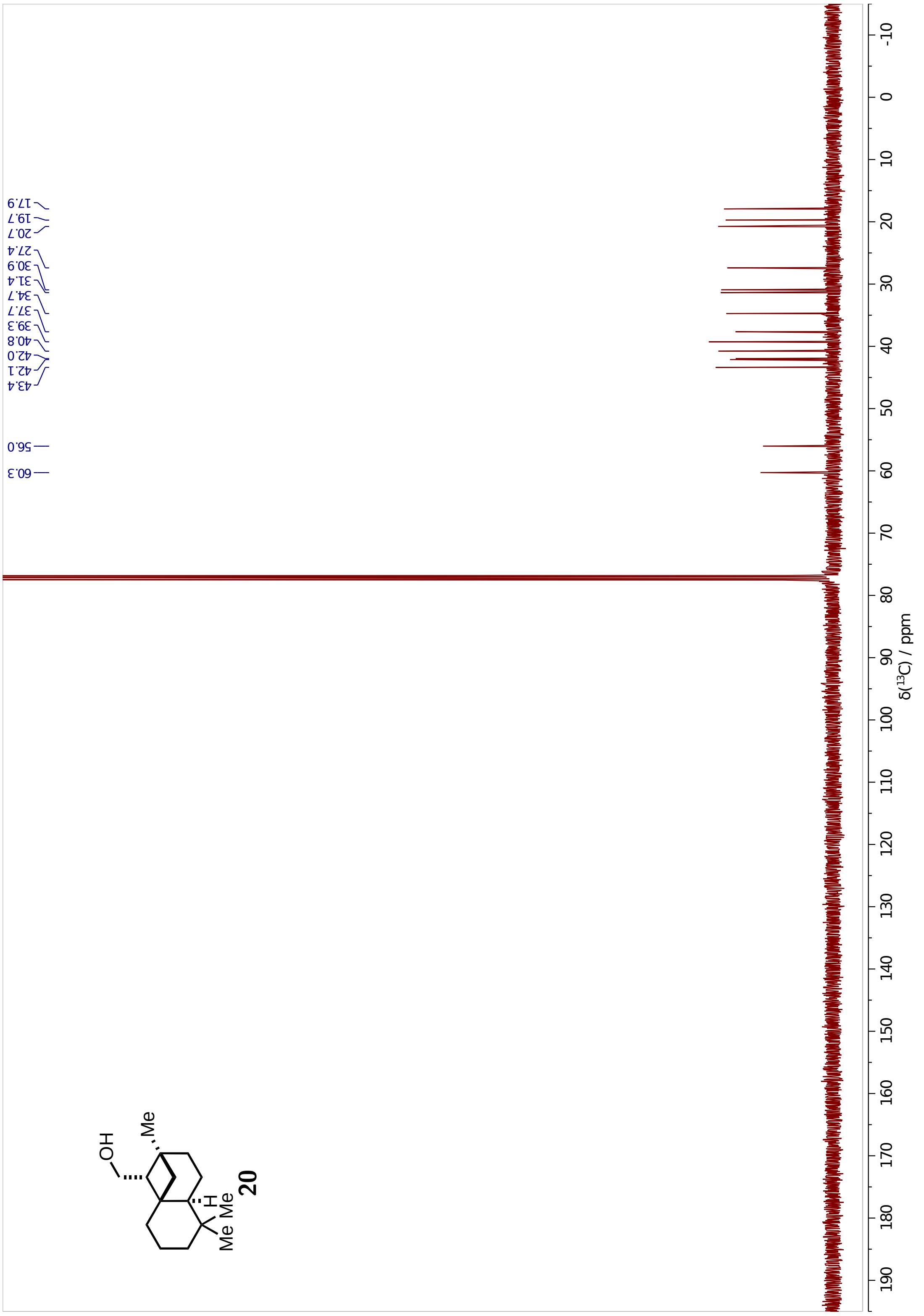


**Spectrum 30** *$^{13}$C-NMR spectrum of substance* ***20*** *measured in $CDCl_3$ at* 101 MHz.

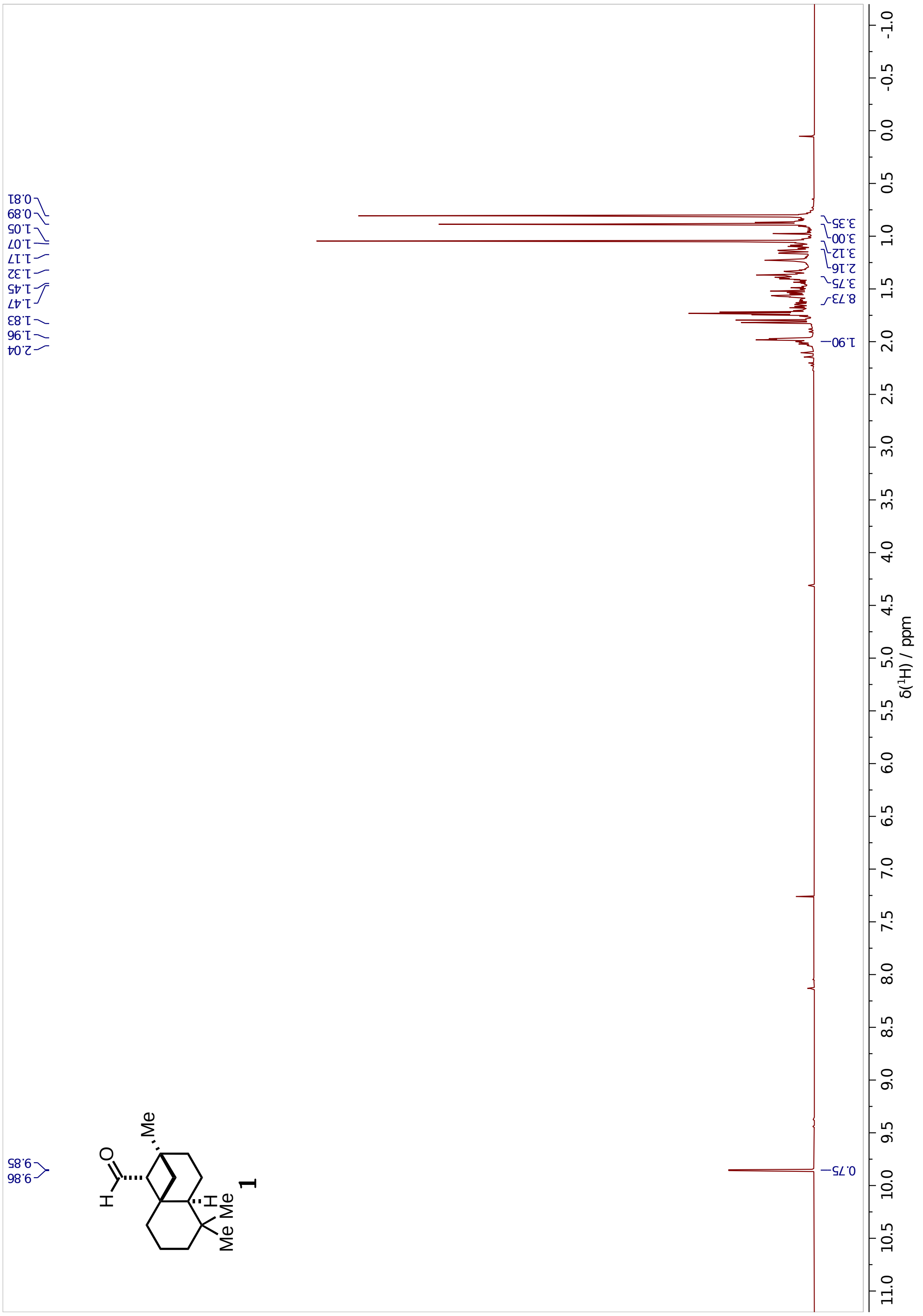


**Spectrum 31** *$^1$H-NMR spectrum of substance **1** measured in $CDCl_3$ at* 400 MHz *at 0 °C.*

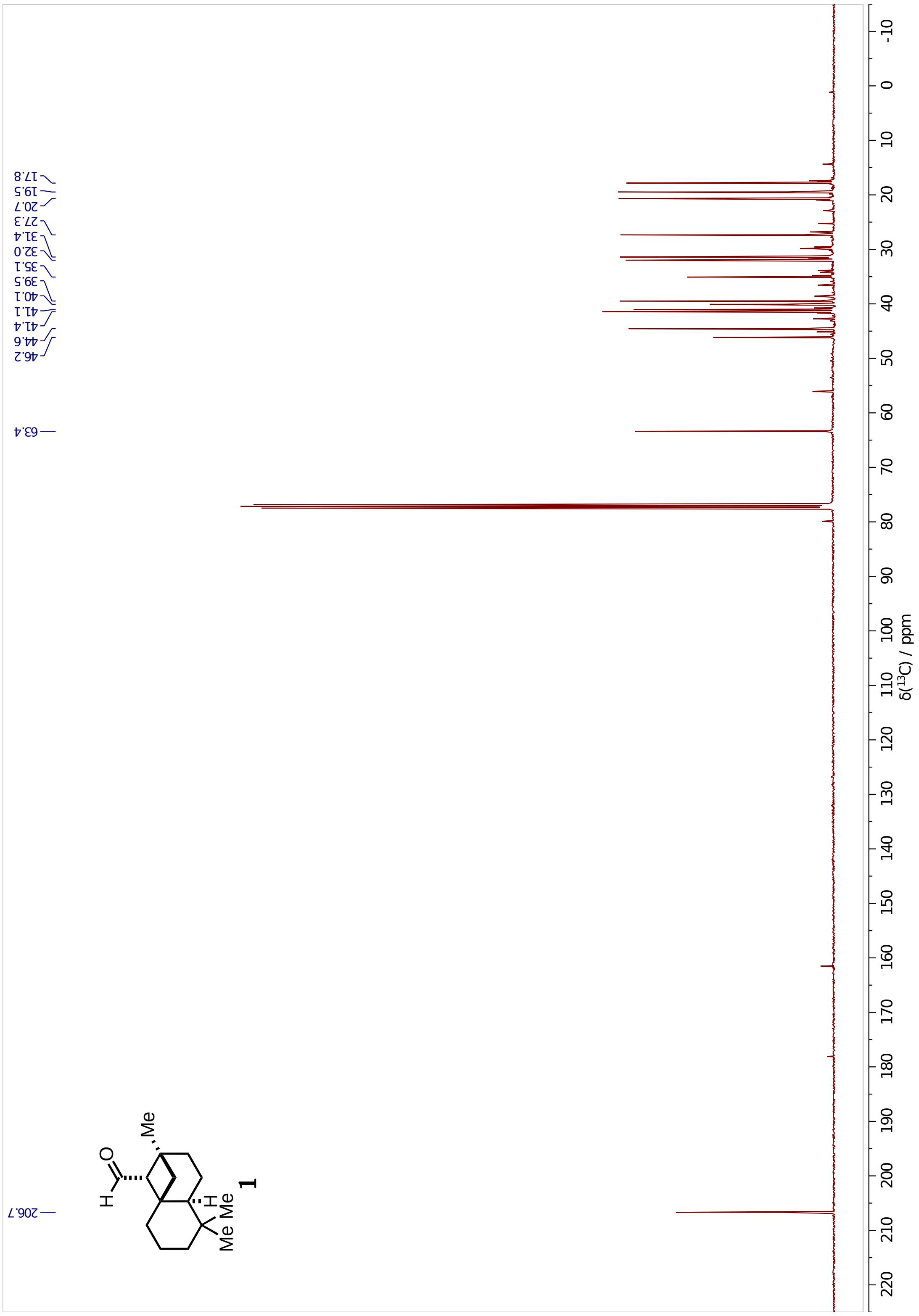


**Spectrum 32** *$^{13}$C-NMR spectrum of substance **1** measured in $CDCl_3$ at* 101 MHz *at 0 °C.*

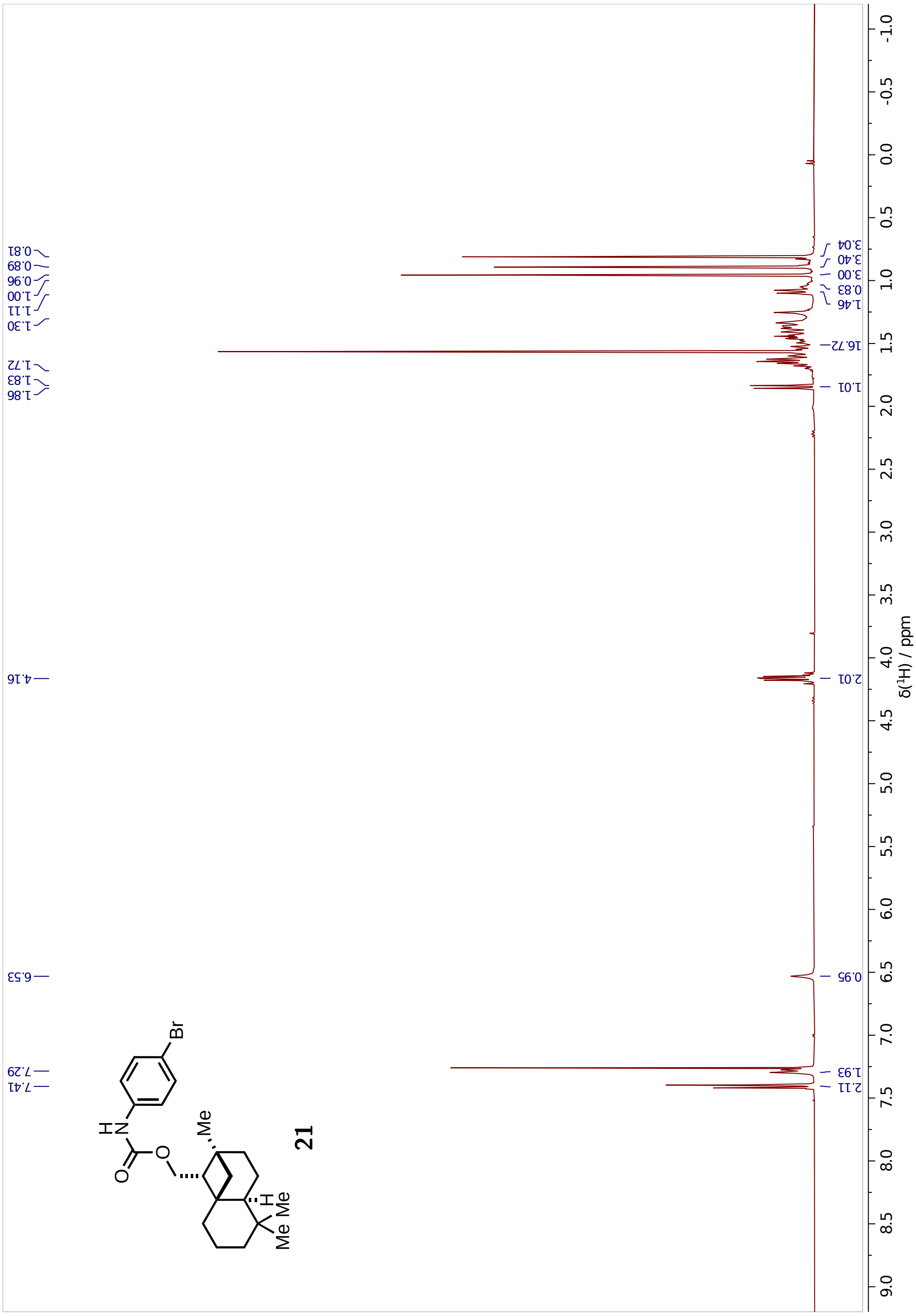


**Spectrum 33** *¹H-NMR spectrum of substance* ***21*** *measured in* $CDCl_3$ *at* 400 MHz.

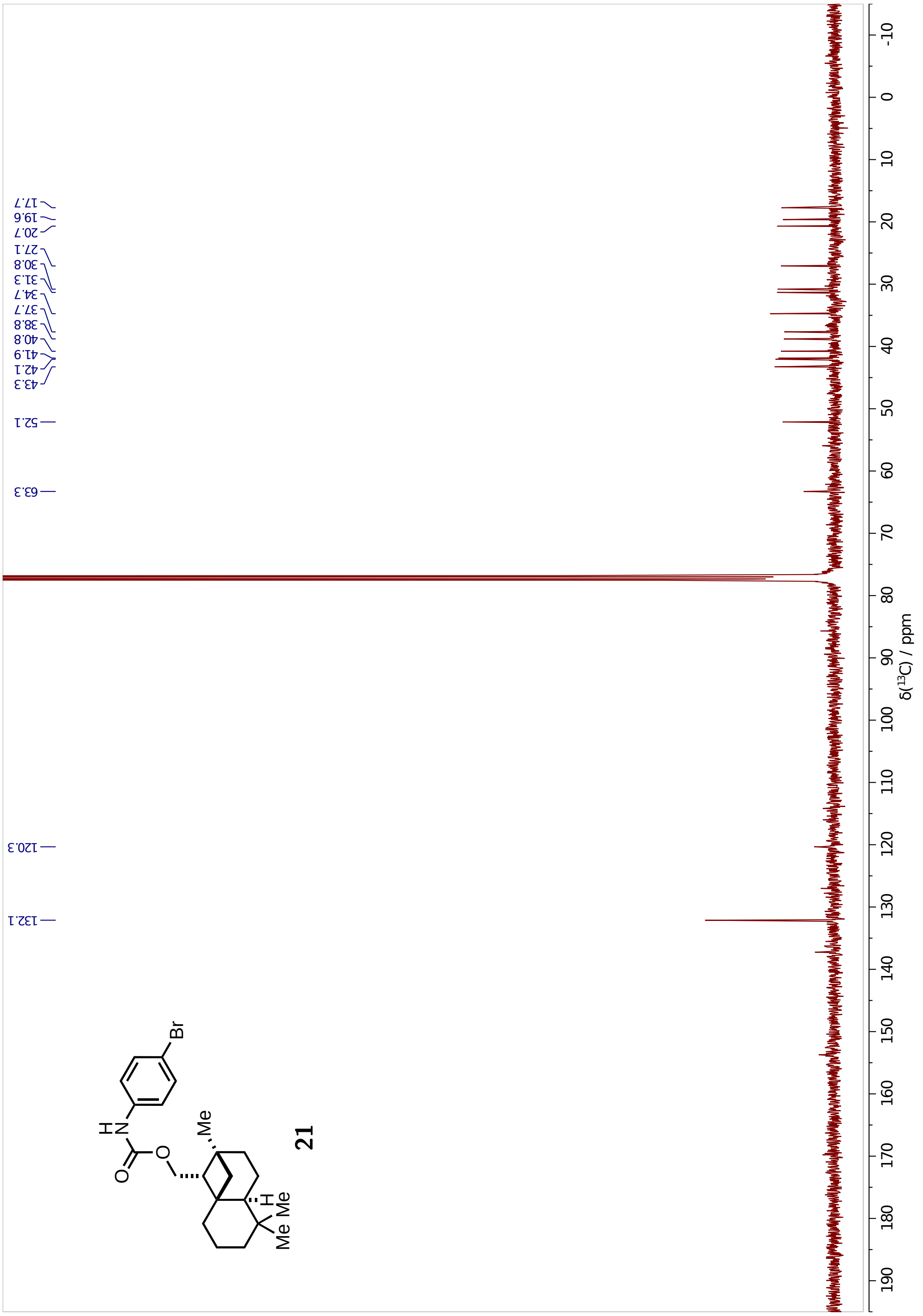


**Spectrum 34** *$^{13}$C-NMR spectrum of substance* ***21*** *measured in $CDCl_3$ at* 101 MHz.
